# Supplementary material for: Leveraging the water-environment-health nexus to characterize sustainable water purification solutions
Source: Nat Commun. 2025 Feb 2;16:1269. doi: 10.1038/s41467-025-56656-6 (PMC11788440; doi:10.1038/s41467-025-56656-6)
Supplement: Supplementary file 1 — Supplementary Information [file 41467_2025_56656_MOESM1_ESM.pdf]

Supplementary Information

Leveraging the water-environment-health nexus to characterize sustainable water purification solutions

Yu-Li Luo[1], Yi-Rong Pan[2,3], Xu Wang[1,4*], Zhao-Yue Wang[1], Glen Daigger[5], Jia-Xin Ma[2], Lin-Hui Tang[1], Junxin Liu[2], Nan-Qi Ren[1], David Butler[4]

[1]State Key Laboratory of Urban Water Resource and Environment, School of Civil and Environmental Engineering, Harbin Institute of Technology, Shenzhen, Shenzhen 518055, China.

[2]Research Center for Eco-Environmental Sciences, Chinese Academy of Sciences, Beijing 100085, China.

[3]International Institute for Applied Systems Analysis, Laxenburg 2361, Austria.

[4]Centre for Water Systems, University of Exeter, Exeter, EX4 4QF, United Kingdom.

[5]Department of Civil and Environmental Engineering, University of Michigan, Ann Arbor, Michigan 48109, United States.

*E-mail: wangxu2021@hit.edu.cn

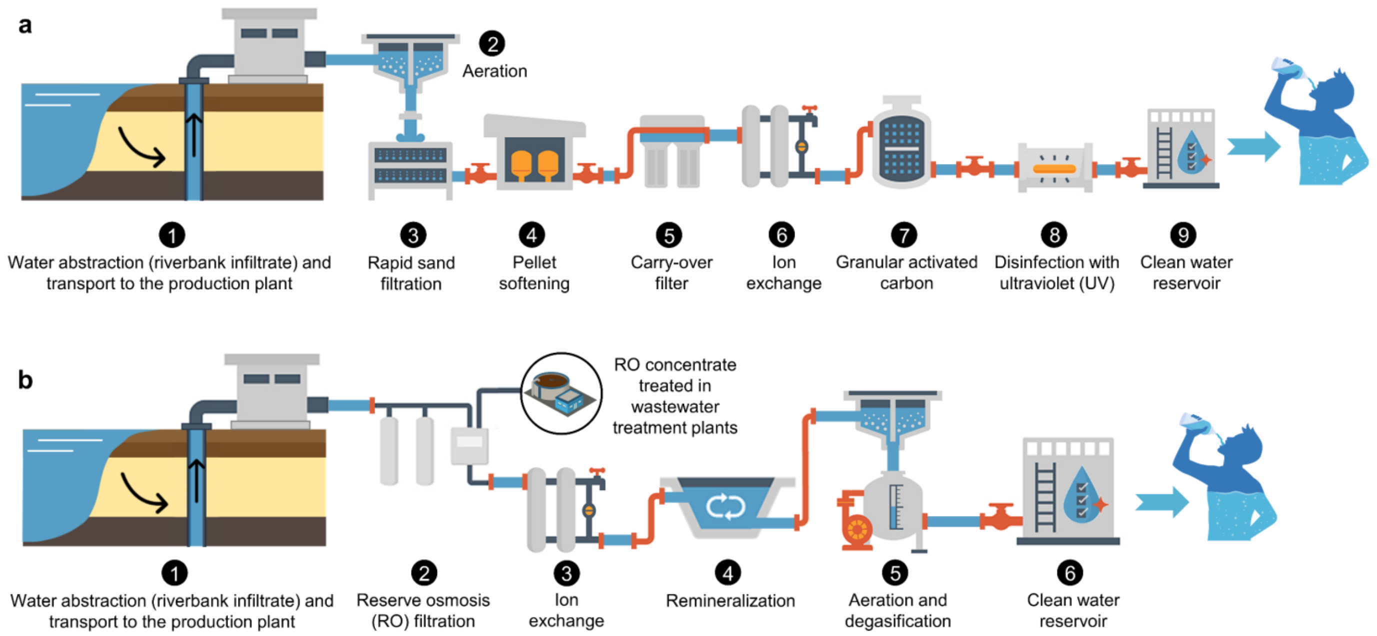

Supplementary Fig. 1 | Process flow diagram illustrating the construction of alternative water production systems. a, riverbank filtration-extended treatment (RBF-ET) system. **b,** riverbank filtration-reserve osmosis (RBF-RO) system. While the management processes for RO concentrate and waste sludge are encompassed within the system boundary of RBF-RO, they are not visualized in the diagram. Water Abstraction (RBF-ET/RBF-RO, Step 1): Groundwater, a combination of surface water and rainwater infiltrated deep into the ground, typically between 15 and 100 meters, requires treatment before it is deemed suitable for drinking. In this study, the extracted water mainly comprises former surface water drawn into the ground via riverbanks. Aeration (RBF-ET, Step 2): Deep groundwater lacks essential oxygen for removing various substances, although it contains other gases such as carbon dioxide and methane. Therefore, extracted water undergoes aeration to oxygenate it and eliminate gases like carbon dioxide and methane before filtration. Rapid Sand Filtration (RBF-ET, Step 3): This step employs sand filters—large containers filled with fine sand—to effectively remove dirt, ensuring bacteriologically reliable water, and addressing issues of iron and manganese. Pellet Softening and Carry-Over Filter (RBF-ET, Steps 4 and 5): To mitigate water hardness caused by calcium and magnesium, a specialized pellet reactor method employing milk of lime or caustic soda facilitates the formation of calcium pellets. Ion Exchange (RBF-ET, Step 6): Anaerobic ion exchange with synthetic fine resins in Na-ionic form further targets residual ammonium removal. Granular Active Carbon (RBF-ET, Step 7): Unlike sand filtration, active carbon filtration uses carbon made from various sources to effectively remove residual substances and traces. Disinfection with UV (RBF-ET, Step 8): Groundwater from bank filtration, having spent years underground, is bacteriologically reliable; however, UV disinfection serves as an additional safety measure by eliminating any remaining bacteria. RO Filtration (RBF-RO, Step 2): Although traditionally thought to remove various substances, it's essential to note that RO membranes do not selectively target pollutants and may require additional treatments for complete purification. Ion Exchange (RBF-RO, Step 3): Despite the effectiveness of RO membranes in removing certain substances, anaerobic ion exchange remains necessary for residual ammonium removal. Remineralization (RBF-RO, Step 4): Post-filtration, the permeate requires remineralization to comply with drinking water regulations and enhance taste. Aeration and Degasification (RBF-RO, Step 5): This step aims to eliminate carbon dioxide and methane dissolved and maintain optimal oxygen saturation levels in the water. Key process parameters of the alternative systems are detailed in Supplementary Table 1.

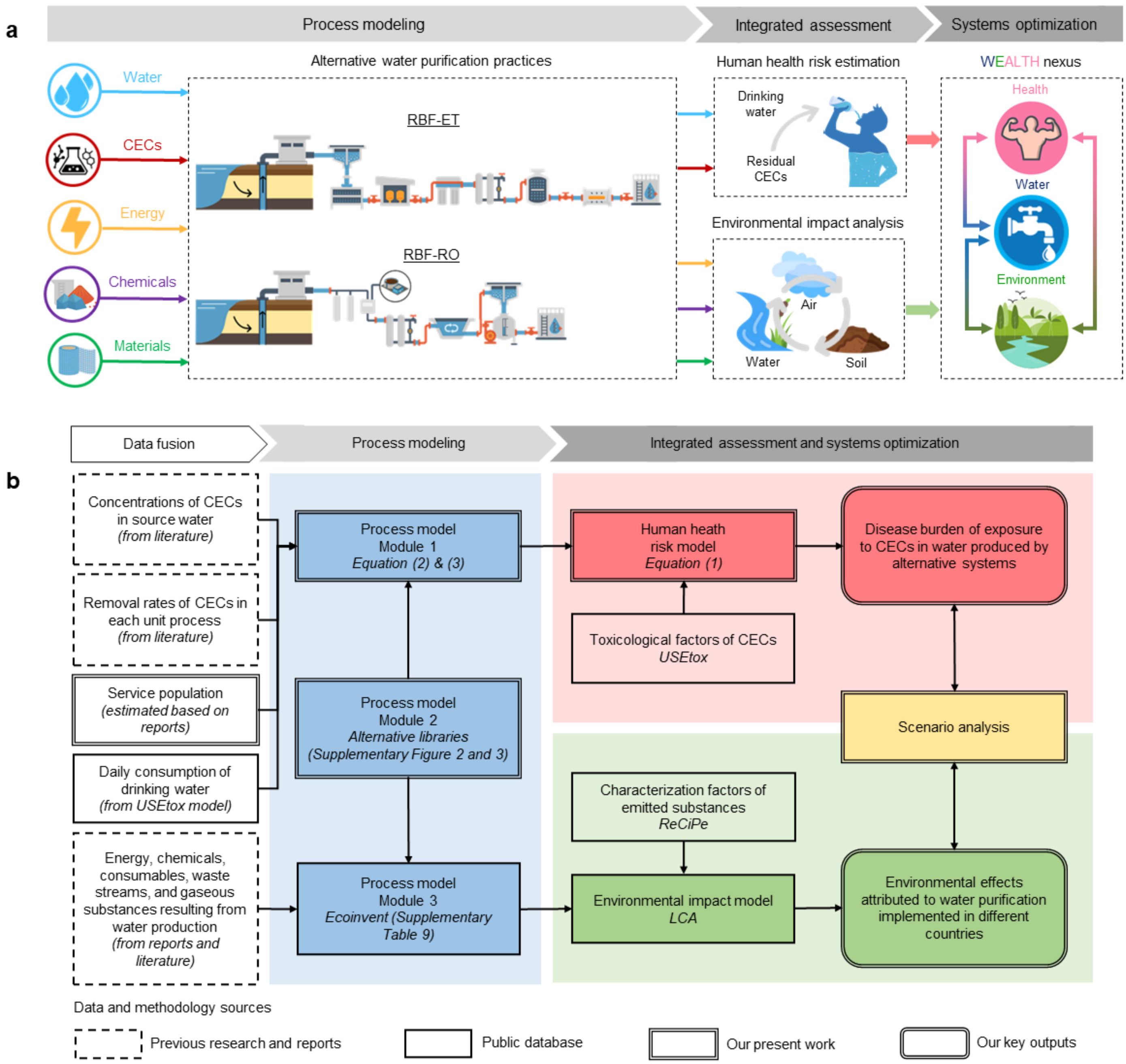

Supplementary Fig. 2 | General approach for characterizing the human health benefits and environmental impacts of water purification solutions. a, Overview of the procedure. **b**, Modeling and integrated analytical framework. Relevant tables, databases, and models are indicated where appropriate.

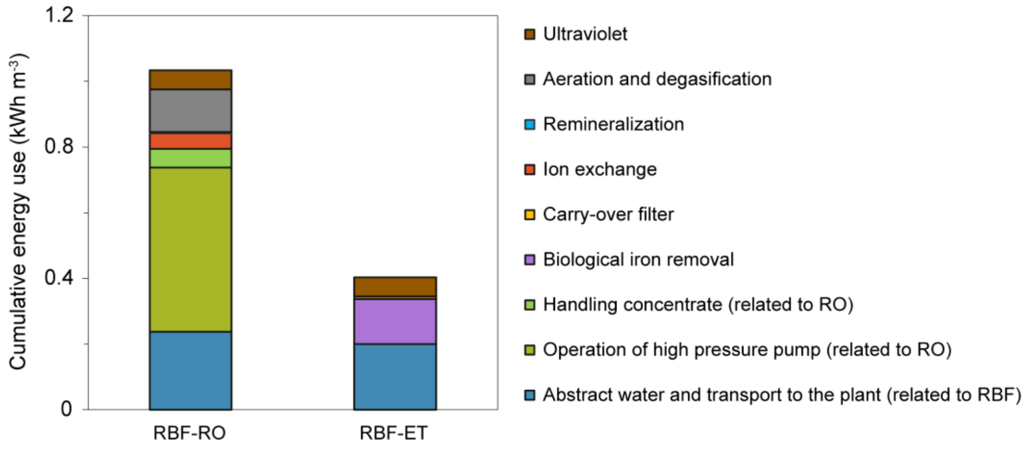

Supplementary Fig. 3 | Cumulative energy use in RBF-RO and RBF-ET systems.

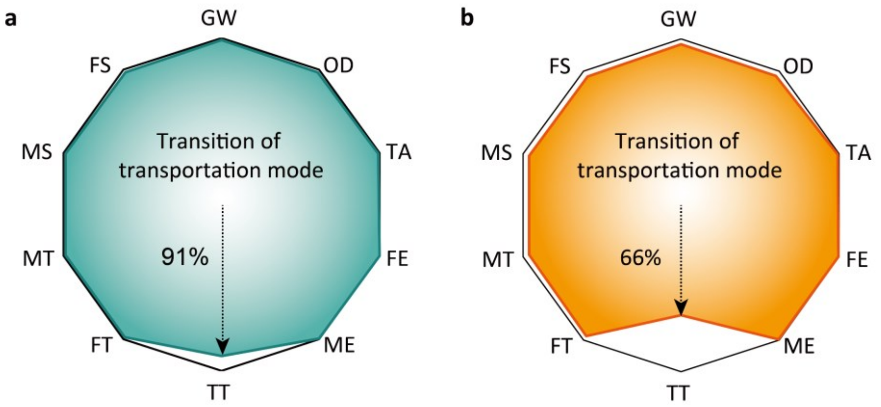

Supplementary Fig. 4 | Reduction in life-cycle environmental impacts resulting from the transition of transportation mode (from trucks to ships). a, RBF-RO system. **b**, RBF-ET system. The black polygon indicates the performance of the original mode (trucks), whereas the colorful polygon represents the performance of the transited mode (ships). Abbreviations: GW, global warming; OD, ozone depletion; TA, terrestrial acidification; FE, freshwater eutrophication; ME, marine eutrophication; TT, terrestrial ecotoxicity; FT, freshwater ecotoxicity; MT, marine ecotoxicity; MC, mineral consumption; FD, fossil fuel depletion.

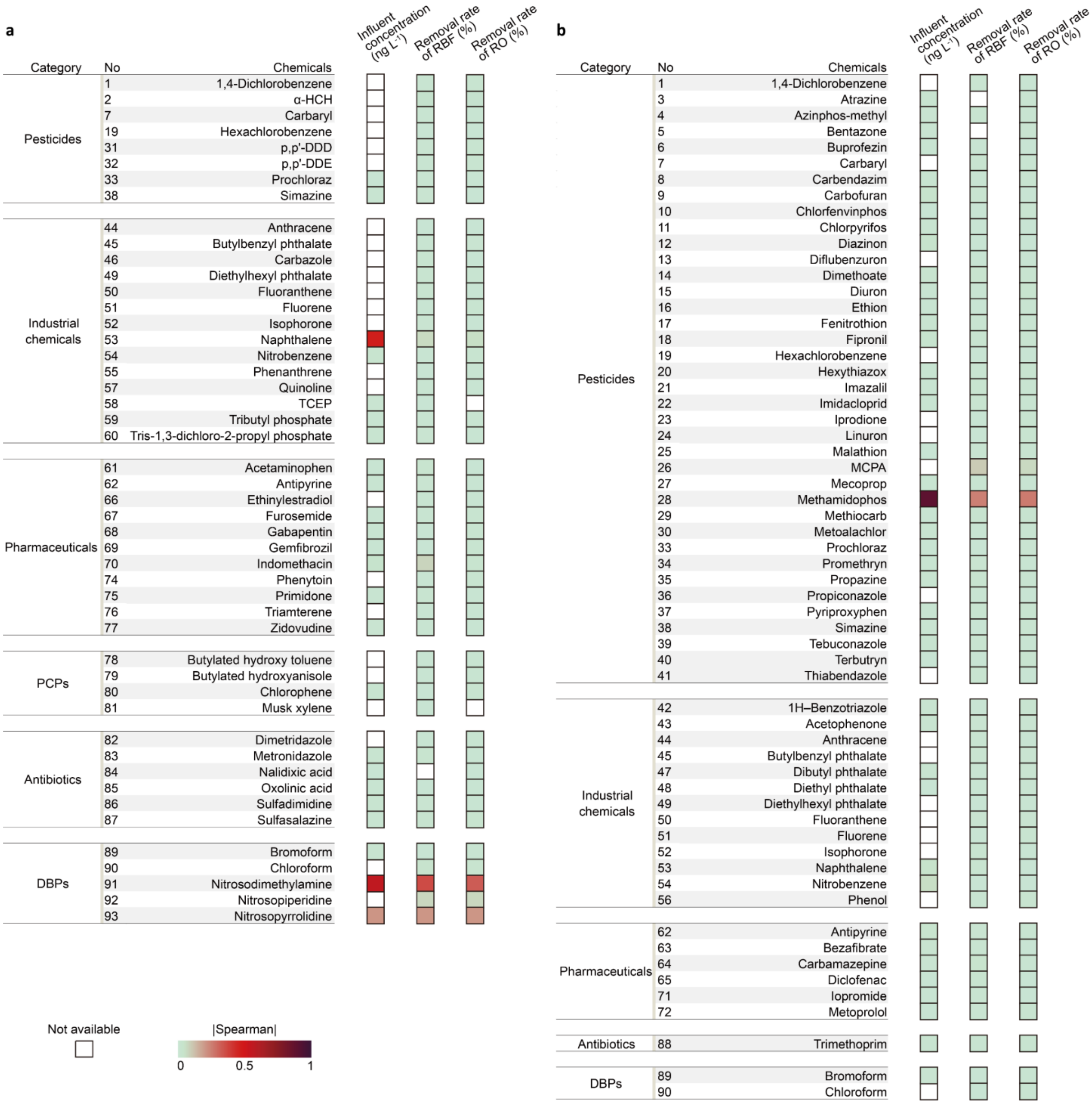

Supplementary Fig. 5 | Sensitivity analysis for influent concentration of CECs in source water and removal rate of RBF and RO configurations. a, Cancer disease burden. **b**, Non-cancer disease burden. The color scale of grids represents the absolute value of Spearman's rank coefficient. The color scale of grids represents the absolute value of Spearman's rank coefficient. The redder the color, the more sensitive the model output is to the input parameter. The white grids indicate that the corresponding parameter was a point value, which was not available for Monte Carlo simulations and sensitivity calculation.

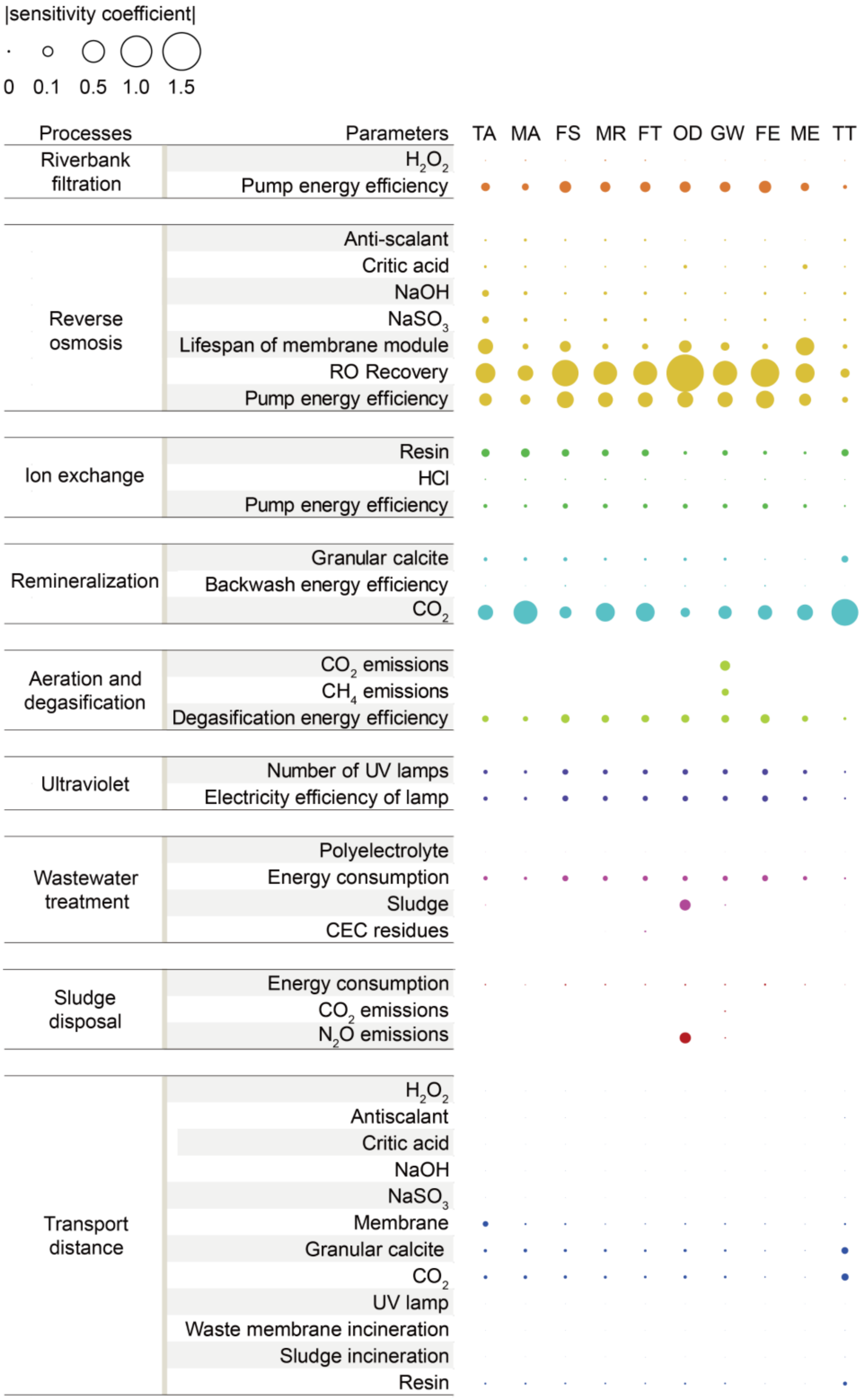

Supplementary Fig.6 | Sensitivity analysis for various processes within the RBF-RO system. The size of each circle corresponds to the absolute value of the sensitivity coefficient, with larger circles indicating higher sensitivity of environmental impacts to the respective process parameter. Circle colors denote different processes relative to the RBF-RO scheme.

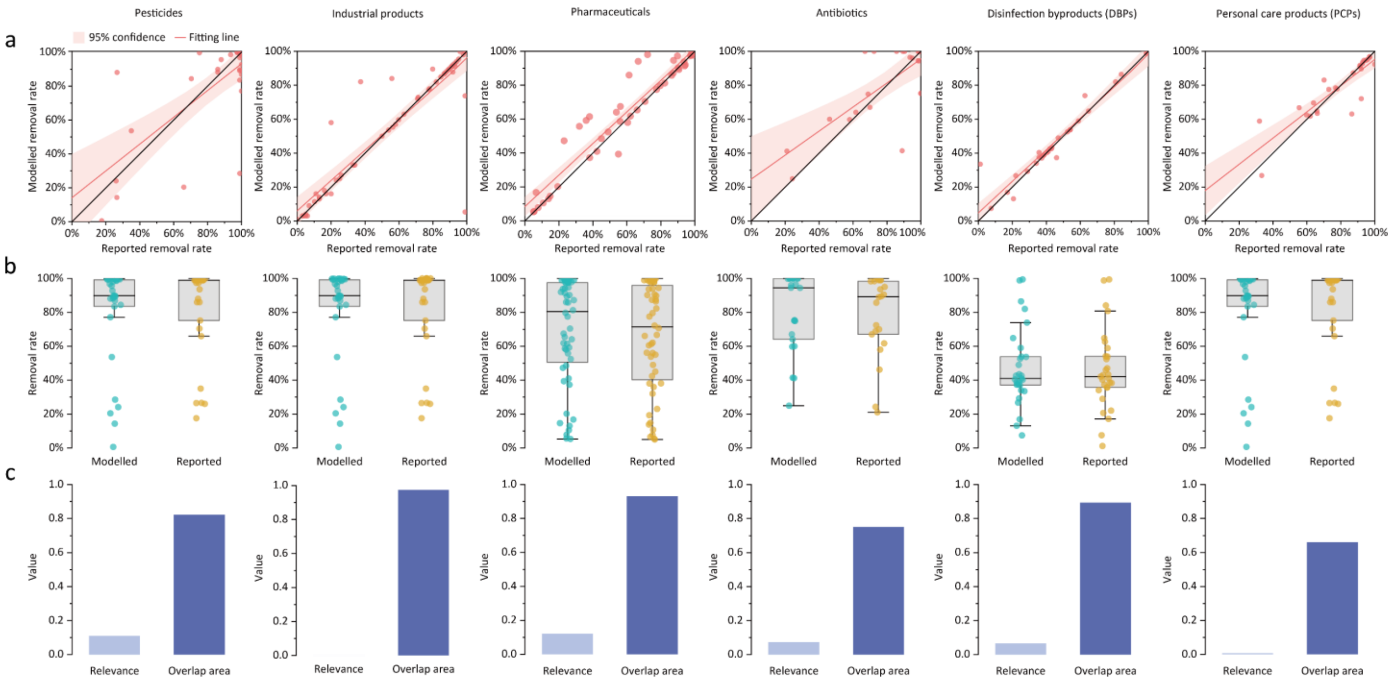

Supplementary Fig. 7 | Evaluation of the reliability of the modelling approach for estimating removal rates of six types of CECs in water treatment processes. **a**, Reported and estimated values for the removal rates of CECs in different treatment configurations. Each pulse circle represents the treatment rate of one individual CEC in a certain treatment process. To assess the universality of the modeling approach, data for 30 pesticides and 3 process configurations, 54 industrial chemicals and 9 process configurations, 98 pharmaceuticals and 10 process configurations, 22 antibiotics and 5 process configurations, 30 DBPs and 3 process configurations, as well as 26 PCPs and 7 process configurations, were derived from the literature and used to prepare the figures. Details with data sources are listed in Supplementary Table 17. It should be noted that both water and wastewater treatment process configurations were included to assess the universality of the model. Pulse lines indicate the average trend, with 95% confidence intervals displayed by pulse shadows, in the relationship of reported and estimated treatment rates of CECs. The modeling approach provides good treatment rate estimations for each type of CECs and overall (pesticides: R2=0.50; industrial chemicals: R^2=0.80; pharmaceuticals: R^2=0.92; antibiotics: R^2=0.51; DBPs: R^2=0.92; PCPs: R^2=0.76; all: R^2=0.95). **b**, Statistical distributions of the reported and modeled removal rates of each type of CECs. The values were derived from the charts in **a**. The center lines represent median values, boxes represent 25th and 75th percentiles, and bars represent 5th to 95th percentiles of the distributions. Each circle indicates the treatment rate of one individual CEC in a certain treatment process. **c**, Uncertainty analysis of the modeling approach by gaining insight into two uncertainty-statistics methods (i.e., impact category relevance, overlap area of probability distributions). Both methods are undertaken by pairwise analysis of the reported and modeled removal rates of CECs derived from the charts in **b**. The impact category relevance is indicated by the absolute value of the mean difference divided by half of the sum of standard deviations. The closer the relevance is to 0, the higher the similarity between the reported and estimated values. The overlap area of probability distributions is represented by the overlap characterization of the fitted probability distributions of reported and modeled removal values. The closer the overlap area is to 1, the higher the degree of fit between the probability distributions of reported and modeled data. In general, the uncertainties derived from the model are acceptable in estimating the treatment rates of CECs.

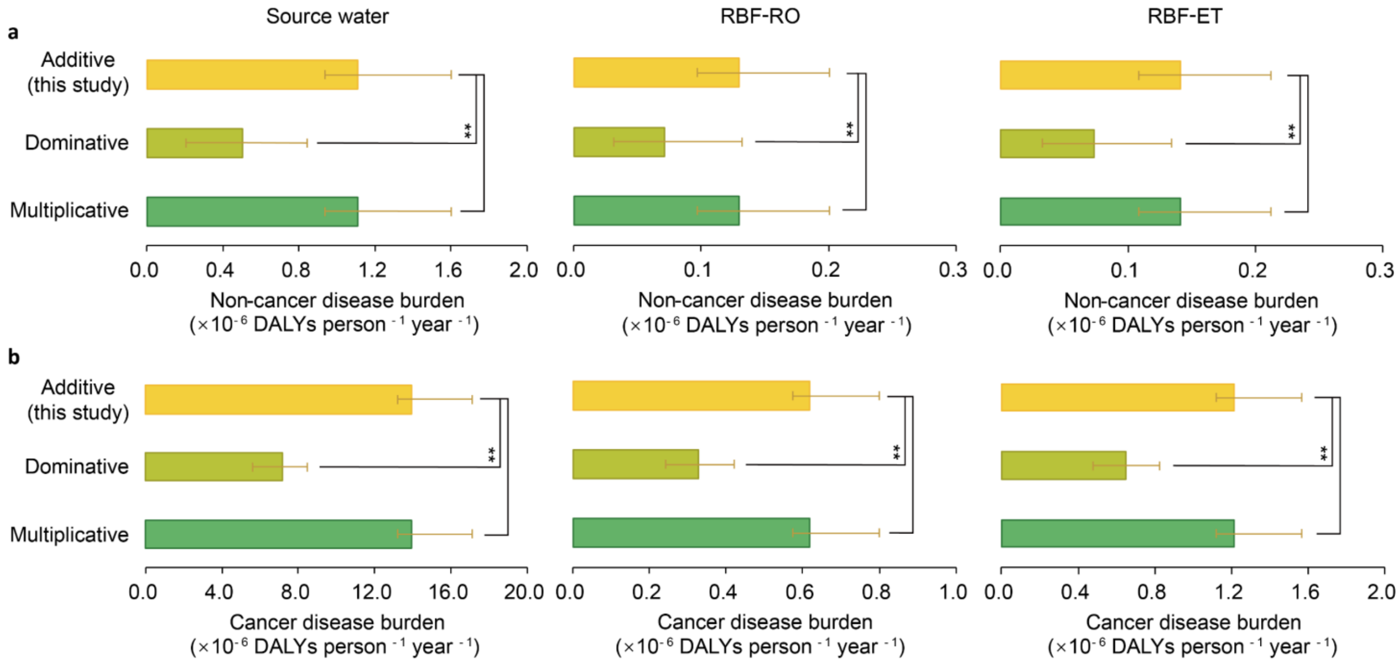

Supplementary Fig. 8 | Comparison of non-cancer (a) and cancer (b) disease burden estimates using alternative mixture toxicity approaches. Data are expressed as mean, with error bars representing the 5th and 95th percentiles. Statistical significance was determined using Student's t-test (** $p < 0.01$ compared with the additive approach used in this study). The calculation procedures associated with the alternative mixture toxicity models are similar with the previous literature[1].

Supplementary Table 1 | Key process parameters of the RBF-RO and RBF-ET systems.		
Process	Category	Key parameter
Riverbank filtration (RBF)	Riverbank filtrate quality	Chemical: H_2O_2 (regeneration of wells)<br>pH: 7.0<br>Temperature: 12 °C<br>Turbidity: <0.1 NTU<br>Total dissolved solids: 750–800 mg L^{-1}<br>Dissolved organic carbon (Humic substances): 8.6 (5.3) mg L^{-1}<br>Calcium: 115.2 mg L^{-1}<br>Sulphate: 43.4 mg L^{-1}<br>Magnesium: 17.4 mg L^{-1}<br>Chloride: 113.6 mg L^{-1}<br>Sodium: 55.2 mg L^{-1}<br>Fluoride: 0.1 mg L^{-1}<br>Potassium: 5.6 mg L^{-1}<br>Bicarbonate: 391.8 mg L^{-1}<br>Barium: 0.1 mg L^{-1}<br>Carbonate: n.a.<br>Strontium: 0.5 mg L^{-1}<br>Nitrate: 0.2 mg L^{-1}<br>Iron (II): 8.5 mg L^{-1}<br>Silica: 16.7 mg L^{-1}<br>Ammonium: 3.7 mg L^{-1}<br>Orthophosphate: 2.1 mg L^{-1}
Extended treatment (ET)	Treatment capacity	340 m^3/h
	Rapid sand filtration	5 units: 2 m bed height, 28 m^2 surface area<br>Residence time: 2.5–5 min<br>Backwash: every 48 hours, 20 min cycle, 250 m^3 volume
	Aeration	2 units: 3 m height, 2.5 m^2 surface area<br>Packing material: Pall-rings, 100 mm<br>Co-current air flow<br>Ratio of air to water flow: 5–10
	Pellet softening	4 units: 5 m height, 1.4 m^2 surface area<br>Residence time: 5.5 min<br>Chemical: NaOH (pH, 8.5–10)<br>Sand grains: 0.15–0.40 mm
	Carry-over filter	3 units: 2 m bed height, 18 m^2 surface area)<br>Hydraulic retention time: 21 min<br>Backwash: every 60 hours, 15 min cycle, 100 m^3
	Ion exchange	Resin type: LEWATIT S 2568 synthetic cation resin<br>2 units: Volume = 1.40 m^3<br>Residence time: 1.5–2.25 min<br>Lifespan: 10 years

	Granular activated carbon filtration	3 units: 2 m bed height, 10 m^2 surface area<br>Residence time: 12–15 min<br>Regeneration: every 37,500 m^3 m^{-3} bed volume
	Disinfection with UV	Medium-pressure UV unit<br>UV dose = 20 mJ cm^{-2}<br>Lamp numbers: 8
	Product water quality	pH: 7.8 ± 0.2<br>Temperature: 11.8 °C<br>Turbidity: <0.1-0.4 FTE<br>Total dissolved solids: 400 ppm<br>Dissolved organic carbon: 6.2 mg L^{-1}<br>Humic substances: 4323 μg-C L^{-1}<br>Calcium: 22.7 mg L^{-1}<br>Sulphate: 50.7 mg L^{-1}<br>Magnesium: 15.5 mg L^{-1}L<br>Chloride: 74.6 mg L^{-1}<br>Sodium: 101.6 mg L^{-1}<br>Fluoride: 0.1 mg L^{-1}<br>Bicarbonate: 219.5 mg L^{-1}<br>Nitrate: 2.89 mg N L^{-1}<br>Iron: 0.01 mg L^{-1}<br>Ammonium (NH^{4+}): <0.02 mg N L^{-1}<br>Phosphate: 0.7 ± 0.1 µg L^{-1} PO_4-P
Reserve osmosis (RO)	Treatment capacity	7 m^3/h
	RO filtration	recovery = 80%<br>3 filtration stages: 9, 3, and 1 pressure vessels<br>3 elements per vessel<br>Membrane surface area = 7.43 m^2<br>Membrane: Hydranautics ESPA2-LD-4040<br>Salt rejection: 99.4%<br>Surface roughness: 89 nm<br>Contact angle: 25-40 o<br>Zeta potential: <-20 mV<br>Membrane molecular weight cut-off values: <200 Da<br>Lifespan: 5 years<br>Flux: 1m^3 h^{-1} $module^{-1}$<br>Feed water pre-treatment: cartridge filter
	Ion exchange	Resin type: LEWATIT S 2568 synthetic cation resin<br>2 units: Volume = 1.40 m^3<br>Residence time: 1.5–2.25 min<br>Lifespan: 10 years
	Remineralization	Calcite contactors<br>Empty bed contact time: 15–20 min

	Aeration and degasification	2 units (Volume = 1.24 m^3)
		Packing material: 38-8 plastic Raflux-Rings, 15 mm
		Counter-current air flow
		Ratio of air to water flow: 10–15
	Product water quality	pH: 7.8 ± 0.2
		Temperature: 12.7 °C
		Turbidity: <0.1–0.3 FTE
		Total dissolved solids: TDS: 150 ppm
		Dissolved organic carbon: <0.3 mg L^{-1}
		Humic substances: 0 μg-C L^{-1}
		Calcium: 40.4 mg L^{-1}
		Magnesium: 3.5 mg L^{-1}
		Fluoride: <0.02 mg L^{-1}
		Bicarbonate: 132.2 mg L^{-1}
		Nitrate: 0.23 mg N L^{-1}
		Ammonium (NH_4^+): <0.02 mg N L^{-1}
		Phosphate: 7.3 ± 0.1 µg L^{-1} PO_4-P

The above-mentioned parameters and values are summarized from the literature[2-4].

Supplementary Table 2 | List of the 93 CECs considered in this analysis.

Category	Chemical	CAS number
Pesticides	1,4-Dichlorobenzene	106-46-7
	α-HCH	608-73-1
	Atrazine	1912-24-9
	Azinphos-methyl	86-50-0
	Bentazone	25057-89-0
	Buprofezin	69327-76-0
	Carbaryl	63-25-2
	Carbendazim	10605-21-7
	Carbofuran	1563-66-2
	Chlorfenvinphos	470-90-6
	Chlorpyrifos	2921-88-2
	Diazinon	333-41-5
	Diflubenzuron	35367-38-5
	Dimethoate	60-51-5
	Diuron	330-54-1
	Ethion	563-12-2
	Fenitrothion	122-14-5
	Fipronil	120068-37-3
	Hexachlorobenzene	118-74-1
	Hexythiazox	78587-05-0
	Imazalil	35554-44-0
	Imidacloprid	138261-41-3
	Iprodione	36734-19-7
	Linuron	330-55-2
	Malathion	121-75-5
	MCPA	94-74-6
	Mecoprop	7085-19-0
	Methamidophos	10265-92-6
	Methiocarb	2032-65-7
	Metoalachlor	51218-45-2
	p,p’-DDD	72-54-8
	p,p’-DDE	72-55-9
	Prochloraz	67747-09-5
	Promethryn	7287-19-6
	Propazine	139-40-2
	Propiconazole	60207-90-1
	Pyriproxyphen	95737-68-1
	Simazine	122-34-9
	Tebuconazole	107534-96-3
	Terbutryn	886-50-0
	Thiabendazole	148-79-8
Industrial chemicals	1H–Benzotriazole	95-14-7
	Acetophenone	98-86-2
	Anthracene	120-12-7
	Butylbenzyl phthalate	81-15-2
	Carbazole	13674-87-8
	Dibutyl phthalate	84-66-2
	Diethyl phthalate	85-68-7
	Diethylhexyl phthalate	84-74-2

	Fluoranthene	206-44-0
	Fluorene	86-73-7
	Isophorone	78-59-1
	Naphthalene	91-20-3
	Nitrobenzene	98-95-3
	Phenanthrene	85-01-8
	Phenol	108-95-2
	Quinoline	91-22-5
	TCEP	126-73-8
	Tributyl phosphate	117-81-7
	Tris-1, 3-dichloro-2-propyl phosphate	115-96-8
Pharmaceuticals	Acetaminophen	103-90-2
	Antipyrine	60-80-0
	Bezafibrate	41859-67-0
	Carbamazepine	298-46-4
	Diclofenac	15307-86-5
	Ethinylestradiol	57-63-6
	Furosemide	54-31-9
	Gabapentin	60142-96-3
	Gemfibrozil	25812-30-0
	Indomethacin	53-86-1
	Iopromide	73334-07-3
	Metoprolol	51384-51-1
	Oxazepam	604-75-1
	Phenytoin	57-41-0
	Primidone	125-33-7
	Triamterene	396-01-0
	Zidovudine	30516-87-1
PCPs	Butylated hydroxy toluene	128-37-0
	Butylated hydroxyanisole	25013-16-5
	Chlorophene	120-32-1
	Musk xylene	81-15-2
Antibiotics	Dimetridazole	551-92-8
	Metronidazole	443-48-1
	Nalidixic acid	389-08-2
	Oxolinic acid	14698-29-4
	Sulfadimidine	57-68-1
	Sulfasalazine	599-79-1
	Trimethoprim	738-70-5
DBPs	Bromoform	75-25-2
	Chloroform	67-66-3
	Nitrosodimethylamine	62-75-9
	Nitrosopiperidine	100-75-4
	Nitrosopyrrolidine	930-55-2

Supplementary Table 3 | Disease burden of exposure to CECs in water after a series of treatments[1].

Process	Category of chemicals	Non-carcinogenic (DALYs person^{-1} year^{-1})			Carcinogenic (DALYs person^{-1} year^{-1})		
		Median	Min	Max	Median	Min	Max
Source water	Pesticides	8.29×10^{-7}	3.62×10^{-7}	1.31×10^{-6}	2.43×10^{-8}	2.14×10^{-8}	2.75×10^{-8}
	Industrial chemicals	1.84×10^{-7}	4.52×10^{-8}	3.25×10^{-7}	1.45×10^{-6}	1.31×10^{-7}	2.78×10^{-6}
	Pharmaceuticals	1.21×10^{-7}	7.55×10^{-8}	1.68×10^{-7}	8.08×10^{-7}	7.66×10^{-7}	8.53×10^{-7}
	PCPs	n/a	n/a	n/a	9.81×10^{-9}	9.77×10^{-9}	9.85×10^{-9}
	Antibiotics	1.91×10^{-9}	1.02×10^{-9}	2.78×10^{-9}	6.25×10^{-9}	5.12×10^{-9}	7.30×10^{-9}
	DBPs	1.26×10^{-7}	1.26×10^{-7}	1.26×10^{-7}	1.29×10^{-5}	1.03×10^{-5}	1.56×10^{-5}
After RBF	Pesticides	4.19×10^{-7}	1.80×10^{-7}	7.63×10^{-7}	1.23×10^{-8}	8.99×10^{-9}	1.57×10^{-8}
	Industrial chemicals	6.64×10^{-8}	1.74×10^{-8}	1.27×10^{-7}	5.34×10^{-7}	4.74×10^{-8}	1.14×10^{-6}
	Pharmaceuticals	3.78×10^{-8}	1.88×10^{-8}	6.33×10^{-8}	1.30×10^{-7}	4.03×10^{-8}	2.31×10^{-7}
	PCPs	n/a	n/a	n/a	3.57×10^{-9}	2.95×10^{-9}	4.19×10^{-9}
	Antibiotics	1.69×10^{-10}	1.44×10^{-11}	4.75×10^{-10}	2.42×10^{-9}	1.72×10^{-9}	3.25×10^{-9}
	DBPs	4.57×10^{-8}	3.70×10^{-8}	5.45×10^{-8}	4.67×10^{-6}	3.44×10^{-6}	6.22×10^{-6}
After RBF-RO	Pesticides	1.35×10^{-7}	6.23×10^{-8}	2.69×10^{-7}	3.60×10^{-9}	2.44×10^{-9}	5.12×10^{-9}
	Industrial chemicals	8.42×10^{-9}	1.96×10^{-9}	1.63×10^{-8}	6.68×10^{-8}	6.34×10^{-9}	1.60×10^{-7}
	Pharmaceuticals	1.48×10^{-9}	7.69×10^{-10}	2.41×10^{-9}	1.58×10^{-8}	4.72×10^{-9}	2.98×10^{-8}
	PCPs	n/a	n/a	n/a	4.13×10^{-10}	3.06×10^{-10}	5.57×10^{-10}
	Antibiotics	8.86×10^{-12}	7.51×10^{-13}	2.80×10^{-11}	3.08×10^{-10}	2.07×10^{-10}	4.29×10^{-10}
	DBPs	5.79×10^{-9}	4.06×10^{-9}	7.90×10^{-9}	5.96×10^{-7}	4.21×10^{-7}	8.38×10^{-7}
After RBF-ET	Pesticides	1.20×10^{-7}	4.96×10^{-8}	2.59×10^{-7}	3.56×10^{-9}	2.43×10^{-9}	5.19×10^{-9}
	Industrial chemicals	1.66×10^{-8}	4.15×10^{-9}	3.24×10^{-8}	1.34×10^{-7}	1.14×10^{-8}	3.14×10^{-7}
	Pharmaceuticals	1.02×10^{-8}	4.63×10^{-9}	2.02×10^{-8}	2.16×10^{-8}	7.70×10^{-9}	4.97×10^{-8}
	PCPs	n/a	n/a	n/a	8.90×10^{-10}	6.54×10^{-10}	1.17×10^{-9}
	Antibiotics	1.31×10^{-11}	1.39×10^{-12}	3.65×10^{-11}	6.05×10^{-10}	4.23×10^{-10}	8.70×10^{-10}
	DBPs	1.14×10^{-8}	7.74×10^{-9}	1.59×10^{-8}	1.17×10^{-6}	8.24×10^{-7}	1.64×10^{-6}

[1] Results are expressed as the overall disease burden associated with each category of CECs. The notation 'n/a' indicates that toxicity data were not applicable for estimating the disease burden. It is important to note that toxicity data for PCPs were only applicable in estimating their carcinogenic effects, as documented in the literature[5].

Supplementary Table 4 | Exposure concentrations, human toxicity factors, and disease burden values of CECs in drinking water processed by the RBF-RO system.

Disease type	Chemical	Chemical Category	Disease burden (DALYs person^{-1} year^{-1})[1]	Exposure concentration (ng L^{-1})[2]	Toxicity factor (cases kg^{-1})
Cancer	Dimetridazole	Antibiotics	1.69×10^{-10}	0.42	6.74×10^{-2}
	Metronidazole		6.16×10^{-12}	0.24	4.03×10^{-3}
	Nalidixic acid		5.46×10^{-11}	0.23	5.70×10^{-3}
	Oxolinic acid		7.93×10^{-11}	1.83	6.86×10^{-3}
	Sulfadimidine		2.71×10^{-12}	0.30	1.35×10^{-3}
	Sulfasalazine		1.68×10^{-11}	1.60	1.63×10^{-3}
	Bromoform	DBPs	7.73×10^{-12}	0.69	1.77×10^{-3}
	Chloroform		1.76×10^{-8}	129.23	2.26×10^{-2}
	Nitrosodimethylamine		3.39×10^{-7}	4.62	12.0
	Nitrosopiperidine		6.97×10^{-8}	7.39	1.57
	Nitrosopyrrolidine		1.91×10^{-7}	10.38	3.01
	Anthracene	Industrial chemicals	6.06×10^{-10}	0.14	0.73
	Butylbenzyl phthalate		1.62×10^{-11}	2.44	1.10×10^{-3}
	Carbazole		3.42×10^{-11}	0.46	1.24×10^{-2}
	Diethylhexyl phthalate		1.22×10^{-10}	6.94	2.92×10^{-3}
	Fluoranthene		4.00×10^{-11}	0.09	7.30×10^{-2}
	Fluorene		1.02×10^{-10}	0.23	7.30×10^{-2}
	Isophorone		5.26×10^{-13}	0.09	9.47×10^{-4}
	Naphthalene		5.40×10^{-8}	78.98	7.30×10^{-2}
	Nitrobenzene		3.15×10^{-9}	7.99	4.49×10^{-2}
	Phenanthrene		1.42×10^{-10}	0.32	7.30×10^{-2}
	Quinoline		2.03×10^{-9}	0.32	1.05
	TCEP		2.94×10^{-10}	1.42	1.32×10^{-2}
	Tributyl phosphate		2.62×10^{-10}	7.27	6.00×10^{-3}
	tris-1,3-dichloro-2-propyl phosphate		2.17×10^{-9}	13.21	2.47×10^{-2}
	Butylated hydroxy toluene	PCPs	4.24×10^{-11}	2.26	3.13×10^{-3}
	Butylated hydroxyanisole		3.71×10^{-10}	21.91	2.83×10^{-3}
	Chlorophene		3.23×10^{-12}	0.33	1.51×10^{-3}
	Musk xylene		3.50×10^{-12}	0.04	1.61×10^{-2}
	1,4-Dichlorobenzene	Pesticides	7.12×10^{-10}	22.81	5.13×10^{-3}
	α-HCH		5.03×10^{-12}	0.01	0.14
	Carbaryl		1.86×10^{-9}	3.68	8.13×10^{-2}
	Hexachlorobenzene		1.08×10^{-11}	0.01	0.30
	p,p'-DDD		6.09×10^{-13}	0.00	6.65×10^{-2}
	p,p'-DDE		5.92×10^{-13}	0.00	0.16
	Prochloraz		6.98×10^{-10}	1.96	5.24×10^{-2}
	Simazine		5.49×10^{-10}	2.68	3.13×10^{-2}
	Acetaminophen	Pharmaceuticals	1.02×10^{-9}	60.80	2.32×10^{-3}
	Antipyrine		7.66×10^{-13}	0.13	9.32×10^{-4}
	Ethinylestradiol		1.57×10^{-9}	0.05	5.73
	Furosemide		6.00×10^{-11}	3.00	2.79×10^{-3}
	Gabapentin		3.50×10^{-12}	1.85	1.96×10^{-4}
	Gemfibrozil		2.37×10^{-12}	0.08	4.64×10^{-3}
	Indomethacin		1.49×10^{-8}	0.12	1.00

	Oxazepam		1.22×10^{-10}	0.35	5.70×10^{-2}
	Phenytoin		1.09×10^{-10}	0.52	3.45×10^{-2}
	Primidone		1.72×10^{-10}	0.36	7.82×10^{-2}
	Triamterene		2.05×10^{-10}	1.01	3.39×10^{-2}
	Zidovudine		4.39×10^{-10}	8.68	6.89×10^{-3}
Non-cancer	Trimethoprim	Antibiotics	8.12×10^{-12}	0.01	2.51×10^{-2}
	Bromoform	DBPs	1.46×10^{-11}	0.69	1.42×10^{-2}
	Chloroform		5.92×10^{-9}	129.23	3.25×10^{-2}
	1H–Benzotriazole	Industrial chemicals	5.26×10^{-10}	16.98	2.11×10^{-2}
	Acetophenone		3.68×10^{-12}	4.11	6.01×10^{-4}
	Anthracene		8.80×10^{-14}	0.14	4.52×10^{-4}
	Butylbenzyl phthalate		5.51×10^{-12}	2.44	1.60×10^{-3}
	dibutyl phthalate		1.07×10^{-9}	334.96	2.03×10^{-3}
	diethyl phthalate		1.94×10^{-11}	37.92	3.39×10^{-4}
	diethylhexyl phthalate		3.99×10^{-10}	6.94	4.07×10^{-2}
	Fluoranthene		4.65×10^{-13}	0.09	3.62×10^{-3}
	Fluorene		1.18×10^{-12}	0.23	3.62×10^{-3}
	Isophorone		7.93×10^{-14}	0.09	6.09×10^{-4}
	Naphthalene		2.24×10^{-9}	78.98	1.29×10^{-2}
	Nitrobenzene		3.63×10^{-9}	7.99	0.22
	Phenol		1.09×10^{-11}	2.35	3.29×10^{-3}
	1,4-Dichlorobenzene	Pesticides	7.27×10^{-11}	22.81	2.23×10^{-3}
	Atrazine		2.30×10^{-9}	5.84	0.25
	Azinphos-methyl		2.19×10^{-9}	1.17	1.24
	Bentazone		6.78×10^{-11}	0.98	1.27×10^{-2}
	Buprofezin		4.31×10^{-12}	0.63	4.38×10^{-3}
	Carbaryl		3.23×10^{-10}	3.68	6.03×10^{-2}
	Carbendazim		4.67×10^{-10}	15.59	1.83×10^{-2}
	Carbofuran		2.94×10^{-9}	3.85	0.42
	Chlorfenvinphos		2.21×10^{-10}	1.19	0.11
	Chlorpyrifos		4.68×10^{-10}	0.91	0.30
	Diazinon		4.24×10^{-9}	4.08	0.64
	Diflubenzuron		2.91×10^{-11}	0.88	2.28×10^{-2}
	Dimethoate		2.28×10^{-9}	4.26	0.31
	Diuron		1.47×10^{-10}	0.91	7.30×10^{-2}
	Ethion		5.18×10^{-10}	3.90	8.47×10^{-2}
	Fenitrothion		2.37×10^{-9}	6.02	0.27
	Fipronil		3.73×10^{-9}	2.94	0.70
	Hexachlorobenzene		8.70×10^{-13}	0.01	0.10
	Hexythiazox		3.48×10^{-11}	1.45	1.52×10^{-2}
	Imazalil		2.66×10^{-10}	8.47	1.83×10^{-2}
	Imidacloprid		9.96×10^{-11}	2.86	2.23×10^{-2}
	Iprodione		8.96×10^{-12}	0.29	2.12×10^{-2}
	linuron		1.91×10^{-10}	0.44	0.29
	Malathion		2.50×10^{-9}	0.63	2.54
	MCPA		2.82×10^{-8}	81.14	0.24
	Mecoprop		3.70×10^{-10}	9.82	2.54×10^{-2}
	Methamidophos		8.89×10^{-8}	69.58	0.78
	Methiocarb		1.91×10^{-11}	0.43	3.04×10^{-2}
	Metoalachlor		1.20×10^{-10}	8.09	8.47×10^{-3}
	Prochloraz		3.05×10^{-10}	1.96	9.77×10^{-2}
	Promethryn		3.08×10^{-13}	0.00	1.22×10^{-2}

Propazine		1.93×10^{-9}	0.96	1.27
Propiconazole		6.79×10^{-12}	0.15	3.18×10^{-2}
Pyriproxyphen		1.25×10^{-11}	1.61	4.57×10^{-3}
Simazine		1.49×10^{-10}	2.68	3.63×10^{-2}
Tebuconazole		3.19×10^{-11}	0.20	1.52×10^{-2}
Terbutryn		3.45×10^{-10}	1.40	0.14
Thiabendazole		1.28×10^{-10}	2.33	3.11×10^{-2}
Antipyrine	Pharmaceuticals	1.92×10^{-12}	0.13	9.92×10^{-3}
Bezafibrate		5.99×10^{-11}	0.96	1.80×10^{-2}
Carbamazepine		2.23×10^{-10}	6.39	2.46×10^{-2}
Diclofenac		1.25×10^{-9}	5.72	0.15
Iopromide		1.26×10^{-11}	3.30	2.06×10^{-3}
Metoprolol		1.98×10^{-12}	0.16	7.19×10^{-3}

[1,2]Median values were used. It should be noted that the toxicity factor, expressed in cases per kilogram, is defined as 50% of the inverse of the ED_{50}. This represents the estimated lifetime ingestion dose that would induce cancer or non-cancer disease in humans, based on calculations of human linear effects. The '50%' refers to the slope factors used in these calculations. The ED_{50}, expressed in kilograms of intake over a lifetime, denotes the lifetime ingestion dose that would induce cancer or non-cancer disease in 50% of a population, as provided by the USEtox model.

Supplementary Table 5 | Contributions of various processes to the terrestrial ecotoxicity associated with truck transportation.

Process	Terrestrial ecotoxicity (kg 1,4-DCB)	Contribution (%)
Diesel, low sulfur	0.0342	2
Lorry, 16 metric ton	0.0545	3
Maintenance, lorry 16 metric ton	0.009129	0
Road	0.0245	1
Brake wear emissions, lorry, wastes	1.97	91
Road wear emissions, lorry, wastes	0	0
Tire wear emissions, lorry, wastes	0.0714	3
Environmental emissions to air	0.0103	0

Supplementary Table 6 | List of 136 countries considered in the context-specific analysis of the RBF-RO system.

Continent	Region	Country[1]	Representativeness[2]	
			Metric A	Metric B
Africa	Eastern	Eritrea, Ethiopia, Kenya, Mauritius, Mozambique, South Sudan, United Republic of Tanzania, Zambia, Zimbabwe	56%	82%
	Middle	Congo, Angola, Congo, Democratic Republic of the Congo, Cameroon, Gabon		
	Northern	Egypt, Algeria, Libya, Morocco, Sudan, Tunisia		
	Southern	South Africa, Botswana, Namibia		
	Western	Benin, Cote d'Ivoire, Ghana, Niger, Nigeria, Senegal, Togo		
Americas	Caribbean	Cuba, Dominican Republic, Haiti, Jamaica, Trinidad and Tobago	69%	100%
	Central	Costa Rica, Guatemala, Honduras, Mexico, Nicaragua, Panama, El Salvador		
	North	Canada, United States		
	South	Argentina, Brazil, Plurinational State of Bolivia, Chile, Colombia, Ecuador, Peru, Paraguay, Uruguay, Venezuela		
Asia	Eastern	China, Republic of Korea, Japan, Democratic People's Republic of Korea, Mongolia	87%	99%
	South-central	India, Bangladesh, Brunei Darussalam, Islamic Republic of Iran, Kyrgyzstan, Kazakhstan, Sri Lanka, Nepal, Pakistan, Tajikistan, Turkmenistan, Uzbekistan		
	South-eastern	Singapore, Vietnam, Indonesia, Cambodia, Myanmar, Malaysia, Philippines, Thailand		
	Western	Turkey, United Arab Emirates, Armenia, Azerbaijan, Bahrain, Cyprus, Georgia, Israel, Iraq, Jordan, Kuwait, Lebanon, Qatar, Saudi Arabia, Syrian Arab Republic, Yemen		
Europe	Eastern	the Czech Republic, Belarus, Hungary, Republic of Moldova, Poland, Romania, Russia, Slovakia, Ukraine	87%	100%
	Northern	Denmark, Estonia, Finland, United Kingdom, Ireland, Iceland, Lithuania, Latvia, Norway, Sweden		
	Southern	Albania, Bosnia and Herzegovina, Spain, Greece, Croatia, Italy, Montenegro, North Macedonia, Malta, Portugal, Serbia, Slovenia		
	Western	Netherlands, Austria, Belgium, Bulgaria, Switzerland, Germany, France, Luxembourg		
Oceania	Australias	New Zealand, Australia	13%	73%

[1] In this analysis, 136 countries across six continents were selected based on the availability of data regarding electricity generation. The representativeness of each set of countries was assessed using two metrics: Metric A, which is the ratio of the total number of selected countries to the total number of countries on that continent, and Metric B, which is the ratio of the total population of the selected countries to the total population of all countries on that continent. Population data for 2017, derived from the UN Database[6], were used for this assessment. Overall, the selected countries account for a majority of the global total, and the total population of these countries comprises more than 90% of the world's population, thereby ensuring a high level of representativeness in this analysis from a global perspective.

Supplementary Table 7 | Airborne emissions associated with generating 1 kWh of electricity from hard coal in coal-rich provinces of India and China.

Substance	Jharkhand, India		Shanxi, China	
	Value	Unit	Value	Unit
Acenaphthene	6.1×10^{-11}	kg	5.0×10^{-11}	kg
Acrolein	3.5×10^{-8}	kg	2.9×10^{-8}	kg
Actinides, radioactive, unspecified	2.5×10^{-4}	kBq	2.1×10^{-4}	kBq
Aldehydes, unspecified	1.1×10^{-7}	kg	9.4×10^{-8}	kg
Antimony	1.7×10^{-8}	kg	1.4×10^{-8}	kg
Arsenic	1.4×10^{-7}	kg	1.2×10^{-7}	kg
Barium	9.4×10^{-7}	kg	7.8×10^{-7}	kg
Benzene	2.0×10^{-6}	kg	1.7×10^{-6}	kg
Benzene, ethyl-	1.1×10^{-8}	kg	9.3×10^{-9}	kg
Benzo(a)pyrene	6.2×10^{-12}	kg	5.2×10^{-12}	kg
Beryllium	2.5×10^{-9}	kg	2.1×10^{-9}	kg
Boron	5.5×10^{-6}	kg	4.6×10^{-6}	kg
Bromine	4.5×10^{-6}	kg	3.8×10^{-6}	kg
Butane	1.6×10^{-7}	kg	1.3×10^{-7}	kg
Cadmium	1.5×10^{-8}	kg	1.2×10^{-8}	kg
Carbon dioxide, fossil	1.4	kg	1.0	kg
Carbon disulfide	1.5×10^{-8}	kg	1.3×10^{-8}	kg
Carbon monoxide, fossil	1.4×10^{-3}	kg	1.1×10^{-4}	kg
Chloroform	1.2×10^{-8}	kg	9.7×10^{-9}	kg
Chromium	1.2×10^{-7}	kg	9.8×10^{-8}	kg
Chromium VI	2.0×10^{-8}	kg	1.7×10^{-8}	kg
Cobalt	4.8×10^{-8}	kg	4.0×10^{-8}	kg
Copper	1.2×10^{-7}	kg	9.9×10^{-8}	kg
Cumene	6.5×10^{-10}	kg	5.4×10^{-10}	kg
Cyanide	3.0×10^{-7}	kg	2.5×10^{-7}	kg
Dinitrogen monoxide	1.9×10^{-5}	kg	1.6×10^{-13}	kg
Dioxin, 2,3,7,8 Tetrachlorodibenzo-p-	1.5×10^{-13}	kg	1.3×10^{-13}	kg
Ethane	3.5×10^{-7}	kg	2.9×10^{-7}	kg
Ethane, 1,1,1-trichloro-, HCFC-140	2.4×10^{-9}	kg	2.0×10^{-9}	kg
Ethane, 1,2-dichloro-	4.8×10^{-9}	kg	3.9×10^{-9}	kg
Ethene, tetrachloro-	5.1×10^{-9}	kg	4.2×10^{-9}	kg
Formaldehyde	5.2×10^{-7}	kg	4.4×10^{-7}	kg
Furan	$6.0E\times10^{-13}$	kg	4.9×10^{-13}	kg
Hexane	8.0×10^{-9}	kg	6.7×10^{-9}	kg
Hydrocarbons, aliphatic, alkanes, cyclic	6.9×10^{-8}	kg	5.7×10^{-8}	kg
Hydrocarbons, aliphatic, alkanes, unspecified	1.9×10^{-6}	kg	1.6×10^{-6}	kg
Hydrocarbons, aliphatic, unsaturated	1.8×10^{-6}	kg	1.5×10^{-6}	kg
Hydrocarbons, chlorinated	2.4×10^{-8}	kg	2.0×10^{-8}	kg
Hydrogen chloride	3.8×10^{-4}	kg	3.2×10^{-4}	kg
Hydrogen fluoride	4.1×10^{-5}	kg	3.4×10^{-5}	kg
Iodine	2.3×10^{-6}	kg	1.9×10^{-6}	kg
Lead	5.4×10^{-7}	kg	4.5×10^{-7}	kg
Lead-210	1.0×10^{-3}	kg	8.3×10^{-4}	kg
Magnesium	1.3×10^{-6}	kg	1.1×10^{-6}	kg
Manganese	5.2×10^{-7}	kg	4.3×10^{-7}	kg
Mercury	3.9×10^{-8}	kg	3.2×10^{-8}	kg
Methane, dichloro-, HCC-30	3.5×10^{-8}	kg	2.9×10^{-8}	kg

Methane, fossil	1.2×10^{-5}	kg	1.1×10^{-5}	kg
Methane, monochloro-, R-40	6.3×10^{-8}	kg	5.2×10^{-8}	kg
Molybdenum	1.7×10^{-8}	kg	1.4×10^{-8}	kg
Nickel	2.9×10^{-7}	kg	2.4×10^{-7}	kg
Nitrogen oxides	2.7×10^{-3}	kg	3.4×10^{-3}	kg
Non-methane volatile organic compounds	1.3×10^{-4}	kg	1.1×10^{-5}	kg
PAH, polycyclic aromatic hydrocarbons	1.1×10^{-7}	kg	9.0×10^{-8}	kg
Particulates, < 2.5 µm	2.5×10^{-3}	kg	3.3×10^{-4}	kg
Particulates, > 10 µm	1.5×10^{-4}	kg	2.4×10^{-3}	kg
Particulates, > 2.5 µm, and < 10 µm	3.0×10^{-4}	kg	5.6×10^{-4}	kg
Pentane	1.3×10^{-6}	kg	1.0×10^{-6}	kg
Phenol	1.9×10^{-9}	kg	1.6×10^{-9}	kg
Polonium-210	1.8×10^{-3}	kBq	1.5×10^{-3}	kBq
Potassium-40	3.3×10^{-4}	kBq	2.8×10^{-4}	kBq
Propane	3.0×10^{-7}	kg	2.5×10^{-7}	kg
Propene	1.4×10^{-7}	kg	1.1×10^{-7}	kg
Protactinium-234	1.5×10^{-5}	kBq	1.2×10^{-5}	kBq
Radium-226	2.6×10^{-4}	kBq	2.2×10^{-4}	kBq
Radium-228	8.5×10^{-5}	kBq	7.1×10^{-5}	kBq
Radon-220	7.3×10^{-3}	kBq	6.1×10^{-3}	kBq
Radon-222	7.2×10^{-3}	kBq	6.0×10^{-3}	kBq
Selenium	2.4×10^{-7}	kg	2.0×10^{-7}	kg
Strontium	8.6×10^{-7}	kg	7.1×10^{-7}	kg
Styrene	2.9×10^{-9}	kg	2.4×10^{-9}	kg
Sulfate	5.7×10^{-9}	kg	4.7×10^{-9}	kg
Sulfur dioxide	2.8×10^{-3}	kg	3.1×10^{-3}	kg
Thorium-228	4.8×10^{-5}	kBq	4.0×10^{-5}	kBq
Thorium-230	1.5×10^{-5}	kBq	1.2×10^{-5}	kBq
Thorium-232	7.0×10^{-5}	kBq	5.8×10^{-5}	kBq
Thorium-234	1.5×10^{-5}	kBq	1.2×10^{-5}	kBq
Toluene	9.6×10^{-7}	kg	8.0×10^{-7}	kg
Uranium-234	2.9×10^{-5}	kBq	2.4×10^{-5}	kBq
Uranium-238	2.3×10^{-4}	kBq	1.9×10^{-4}	kBq
Vanadium	2.1×10^{-7}	kg	1.8×10^{-7}	kg
Water	2.1×10^{-3}	m^3	1.7×10^{-3}	m^3
Xylene	7.9×10^{-6}	kg	6.6×10^{-6}	kg
Zinc	6.0×10^{-7}	kg	5.0×10^{-7}	kg

Supplementary Table 8 | Comparative analysis of airborne emissions during electricity generation from lignite, hard coal, and natural gas in Australia.

Substance	Hard coal		Lignite		Natural gas	
	Value	Unit	Value	Unit	Value	Unit
Acenaphthene	4.6×10^{-11}	kg	n/a	kg	5.4×10^{-12}	kg
Acetaldehyde	n/a	kg	n/a	kg	5.5×10^{-9}	kg
Acetic acid	n/a	kg	n/a	kg	8.2×10^{-7}	kg
Acrolein	2.6×10^{-8}	kg	n/a	kg	n/a	kg
Actinides, radioactive, unspecified	1.9×10^{-4}	kBq	n/a	kBq	n/a	kBq
Aldehydes, unspecified	8.5×10^{-8}	kg	n/a	kg	n/a	kg
Antimony	1.3×10^{-8}	kg	5.8×10^{-9}	kg	n/a	kg
Arsenic	1.1×10^{-7}	kg	3.3×10^{-8}	kg	2.8×10^{-10}	kg
Barium	7.1×10^{-7}	kg	1.7×10^{-7}	kg	n/a	kg
Benzene	1.5×10^{-6}	kg	2.4×10^{-6}	kg	6.1×10^{-9}	kg
Benzene, ethyl-	8.4×10^{-9}	kg	n/a	kg	n/a	kg
Benzo(a)pyrene	4.7×10^{-12}	kg	2.2×10^{-12}	kg	3.6×10^{-12}	kg
Beryllium	1.9×10^{-9}	kg	n/a	kg	1.7×10^{-11}	kg
Boron	4.2×10^{-6}	kg	2.1×10^{-5}	kg	n/a	kg
Bromine	3.4×10^{-6}	kg	7.4×10^{-7}	kg	n/a	kg
Butane	1.2×10^{-7}	kg	2.1×10^{-7}	kg	6.3×10^{-6}	kg
Cadmium	1.1×10^{-8}	kg	7.5×10^{-9}	kg	1.5×10^{-9}	kg
Carbon dioxide, fossil	1.0	kg	1.2	kg	0.36	kg
Carbon disulfide	1.2×10^{-8}	kg	n/a	kg	n/a	kg
Carbon monoxide, fossil	9.6×10^{-5}	kg	2.2×10^{-4}	kg	1.5×10^{-5}	kg
Chloroform	8.8×10^{-9}	kg	n/a	kg	n/a	kg
Chromium	8.9×10^{-8}	kg	3.0×10^{-8}	kg	2.0×10^{-9}	kg
Chromium VI	1.5×10^{-8}	kg	3.6×10^{-9}	kg	n/a	kg
Cobalt	3.6×10^{-8}	kg	6.0×10^{-9}	kg	1.2×10^{-10}	kg
Copper	8.9×10^{-8}	kg	8.3×10^{-8}	kg	n/a	kg
Cumene	4.9×10^{-10}	kg	n/a	kg	n/a	kg
Cyanide	2.2×10^{-7}	kg	n/a	kg	n/a	kg
Dinitrogen monoxide	1.4×10^{-5}	kg	1.60×10^{-5}	kg	6.7×10^{-6}	kg
Dioxin, 2,3,7,8 Tetrachlorodibenzo-p-	1.2×10^{-13}	kg	7.8×10^{-14}	kg	n/a	kg
Ethane	2.6×10^{-7}	kg	4.6×10^{-7}	kg	9.3×10^{-6}	kg
Ethane, 1,1,1-trichloro-, HCFC-140	1.8×10^{-9}	kg	n/a	kg	n/a	kg
Ethane, 1,2-dichloro-	3.6×10^{-9}	kg	n/a	kg	n/a	kg
Ethene, tetrachloro-	3.9×10^{-9}	kg	n/a	kg	n/a	kg
Formaldehyde	3.9×10^{-7}	kg	6.5×10^{-7}	kg	2.2×10^{-7}	kg
Furan	4.5×10^{-13}	kg	n/a	kg	n/a	kg
Hexane	6.0×10^{-9}	kg	n/a	kg	5.4×10^{-6}	kg
Hydrocarbons, aliphatic, alkanes, cyclic	5.2×10^{-8}	kg	n/a	kg	n/a	kg
Hydrocarbons, aliphatic, alkanes, unspecified	1.5×10^{-6}	kg	2.4×10^{-6}	kg	n/a	kg
Hydrocarbons, aliphatic, unsaturated	1.4×10^{-6}	kg	2.4×10^{-6}	kg	n/a	kg
Hydrocarbons, chlorinated	1.8×10^{-8}	kg	n/a	kg	n/a	kg
Hydrogen chloride	2.9×10^{-4}	kg	6.3×10^{-5}	kg	n/a	kg
Hydrogen fluoride	3.1×10^{-5}	kg	1.5×10^{-5}	kg	n/a	kg
Iodine	1.7×10^{-6}	kg	5.4×10^{-7}	kg	n/a	kg
Lead	4.0×10^{-7}	kg	2.9×10^{-8}	kg	7.0×10^{-10}	kg
Lead-210	7.5×10^{-4}	kBq	9.4×10^{-5}	kBq	n/a	kBq
Magnesium	9.9×10^{-7}	kg	n/a	kg	n/a	kg

Manganese	3.9×10^{-7}	kg	9.5×10^{-8}	kg	5.3×10^{-10}	kg
Mercury	2.9×10^{-8}	kg	6.0×10^{-8}	kg	4.7×10^{-10}	kg
Methane, dichloro-, HCC-30	2.6×10^{-8}	kg	n/a	kg	n/a	kg
Methane, fossil	9.8×10^{-6}	kg	1.1×10^{-5}	kg	6.6×10^{-6}	kg
Methane, monochloro-, R-40	4.8×10^{-8}	kg	n/a	kg	n/a	kg
Molybdenum	1.3×10^{-8}	kg	1.8×10^{-8}	kg	n/a	kg
Nickel	2.2×10^{-7}	kg	1.1×10^{-7}	kg	2.9×10^{-9}	kg
Nitrogen oxides	2.5×10^{-3}	kg	1.9×10^{-3}	kg	1.7×10^{-4}	kg
Non-methane volatile organic compounds	1.0×10^{-5}	kg	n/a	kg	n/a	kg
PAH, polycyclic aromatic hydrocarbons	8.1×10^{-8}	kg	1.1×10^{-8}	kg	5.5×10^{-8}	kg
Particulates, < 2.5 µm	6.8×10^{-5}	kg	6.7×10^{-5}	kg	3.3×10^{-6}	kg
Particulates, > 10 µm	3.8×10^{-5}	kg	4.9×10^{-5}	kg	n/a	kg
Particulates, > 2.5 µm, and < 10 µm	7.6×10^{-5}	kg	9.8×10^{-5}	kg	n/a	kg
Pentane	9.5×10^{-7}	kg	1.6×10^{-6}	kg	7.8×10^{-6}	kg
Phenol	1.4×10^{-9}	kg	n/a	kg	n/a	kg
Polonium-210	1.3×10^{-3}	kBq	1.7×10^{-4}	kBq	n/a	kBq
Potassium-40	2.5×10^{-4}	kg	3.2×10^{-5}	kg	n/a	kg
Propane	2.3×10^{-7}	kg	3.9×10^{-7}	kg	4.8×10^{-6}	kg
Propene	1.0×10^{-7}	kg	1.8×10^{-7}	kg	n/a	kg
Propionic acid	n/a	kg	n/a	kg	1.1×10^{-7}	kg
Protactinium-234	1.1×10^{-5}	kBq	n/a	kBq	n/a	kBq
Radium-226	2.0×10^{-4}	kBq	2.4×10^{-5}	kBq	n/a	kBq
Radium-228	6.4×10^{-5}	kBq	1.4×10^{-5}	kBq	n/a	kBq
Radon-220	5.5×10^{-3}	kBq	1.4×10^{-3}	kBq	n/a	kBq
Radon-222	5.4×10^{-3}	kBq	2.5×10^{-3}	kBq	n/a	kBq
Selenium	1.8×10^{-7}	kg	1.4×10^{-7}	kg	3.3×10^{-11}	kg
Strontium	6.4×10^{-7}	kg	1.5×10^{-7}	kg	n/a	kg
Styrene	2.2×10^{-9}	kg	n/a	kg	n/a	kg
Sulfate	4.3×10^{-9}	kg	n/a	kg	n/a	kg
Sulfur dioxide	3.6×10^{-3}	kg	2.6×10^{-3}	kg	3.9×10^{-6}	kg
Thorium-228	3.6×10^{-5}	kg	7.8×10^{-6}	kg	n/a	kg
Thorium-230	1.1×10^{-5}	kg	n/a	kg	n/a	kg
Thorium-232	5.3×10^{-5}	kg	1.2×10^{-5}	kg	n/a	kg
Thorium-234	1.1×10^{-5}	kg	n/a	kg	n/a	kg
Toluene	7.2×10^{-7}	kg	1.2×10^{-6}	kg	1.0×10^{-8}	kg
Uranium-234	2.2×10^{-5}	kg	n/a	kg	n/a	kg
Uranium-238	1.7×10^{-4}	kg	2.0×10^{-5}	kg	n/a	kg
Vanadium	1.6×10^{-7}	kg	3.8×10^{-8}	kg	n/a	kg
Water	1.6×10^{-3}	m^3	1.5×10^{-3}	m^3	6.7×10^{-4}	m^3
Xylene	5.9×10^{-6}	kg	1.0×10^{-5}	kg	n/a	kg
Zinc	4.5×10^{-7}	kg	1.9×10^{-7}	kg	n/a	kg

Supplementary Table 9 | Concentrations of 93 CECs in source water[1].

Category	No	Chemicals	Median	Min	Max	Reference
Pesticides	1	1,4-Dichlorobenzene	155	n/a	n/a	7,8
	2	α-HCH	0.04	n/a	n/a	8
	3	Atrazine	23	17	33	7-18
	4	Azinphos-methyl	8	7	9	15,16
	5	Bentazone	28	10	51	12,19
	6	Buprofezin	4	3	6	15,16
	7	Carbaryl	25	n/a	n/a	7
	8	Carbendazim	102	26	198	8,9,15,16
	9	Carbofuran	22	4	56	9,12,15
	10	Chlorfenvinphos	8	2	15	15,16
	11	Chlorpyrifos	6	2	11	15-18
	12	Diazinon	27	14	40	15-18
	13	Diflubenzuron	6	n/a	n/a	8
	14	Dimethoate	20	5	37	12,15,16,18
	15	Diuron	39	28	49	7-9,15,16,18
	16	Ethion	27	14	40	15
	17	Fenitrothion	41	35	47	15,16
	18	Fipronil	17	2	44	20
	19	Hexachlorobenzene	0.04	n/a	n/a	8
	20	Hexythiazox	10	3	17	15,16
	21	Imazalil	49	6	127	15,16
	22	Imidacloprid	19	13	26	7,9,15,16
	23	Iprodione	2	n/a	n/a	8
	24	Linuron	3	n/a	n/a	8,9,11,17,18
	25	Malathion	4	3	6	15,16
	26	MCPA	447	n/a	n/a	18
	27	Mecoprop	96	89	103	18
	28	Methamidophos	471	57	923	12
	29	Methiocarb	3	3	3	9,15,16
	30	Metoalachlor	47	12	113	7,11,12,14-16
	31	p,p'-DDD	0.01	n/a	n/a	8
	32	p,p'-DDE	0.004	n/a	n/a	8
	33	Prochloraz	13	6	22	8,15,16
	34	Promethryn	0.2	n/a	n/a	9
	35	Propazine	6	5	9	9,15,16
	36	Propiconazole	n/a	0	1	9,19
	37	Pyriproxyphen	12	1	20	15,16
	38	Simazine	18	13	24	9,12,15-18
	39	Tebuconazole	n/a	2	15	16
	40	Terbutryn	9	2	18	9,15,16
	41	Thiabendazole	14	3	32	15,16
Industrial chemicals	42	1H–Benzotriazole	389	282	532	21
	43	Acetophenone	87	57	125	7,8
	44	Anthracene	3	n/a	n/a	8
	45	Butylbenzyl phthalate	n/a	14	41	11
	46	Carbazole	150	87	214	8
	47	Dibutyl phthalate	836	282	1376	22
	48	Diethyl phthalate	53	n/a	n/a	8,22

	49	Diethylhexyl phthalate	7282	737	13907	11
	50	Fluoranthene	2	n/a	n/a	8
	51	Fluorene	5	n/a	n/a	8
	52	Isophorone	2	n/a	n/a	8
	53	Naphthalene	17	0	5895	8
	54	Nitrobenzene	40	0	548	8
	55	Phenanthrene	7	n/a	n/a	8
	56	Phenol	51	n/a	n/a	7,8
	57	Quinoline	7	n/a	n/a	8
	58	TCEP	158	153	163	11,17,23
	59	Tributyl phosphate	150	n/a	n/a	7,18
	60	Tris-1,3-dichloro-2-propyl phosphate	149	74	225	23
Pharmaceuticals	61	Acetaminophen	2589	497	4635	18-20,24-32
	62	Antipyrine	4	2	6	30
	63	Bezafibrate	278	67	493	26,27,29,32-35
	64	Carbamazepine	197	67	328	7,9-11,17,19,24-32,34-41
	65	Diclofenac	515	300	738	7,9-11,18,24,26-29,31-33,35,42-46
	66	Ethinylestradiol	1	n/a	n/a	21,26
	67	Furosemide	83	32	171	19,33
	68	Gabapentin	497	272	726	7,33
	69	Gemfibrozil	15	9	20	7,9-11,17,24,27,29,31,35,41,43,44,46
	70	Indomethacin	117	38	196	30,35,44,46
	71	Iopromide	311	44	566	10,24,35,36
	72	Metoprolol	54	26	81	7,26,29,31,33,35,38
	73	Oxazepam	11	9	12	19,26,37
	74	Phenytoin	16	n/a	n/a	7,11,17
	75	Primidone	n/a	5	6	10,17,30
	76	Triamterene	31	n/a	n/a	7,17
	77	Zidovudine	n/a	220	620	32
PCPs	78	Butylated hydroxy toluene	49	n/a	n/a	11
	79	Butylated hydroxyanisole	475	n/a	n/a	7
	80	Chlorophene	7	3	12	33
	81	Musk xylene	10	n/a	n/a	35,44
Antibiotics	82	Dimetridazole	9	n/a	n/a	29,31
	83	Metronidazole	5	3	8	26,29,31,33,47
	84	Nalidixic acid	17	6	27	29,32,35,46,48
	85	Oxolinic acid	40	21	59	15,35,48
	86	Sulfadimidine	6	3	11	49
	87	Sulfasalazine	34	16	55	33
	88	Trimethoprim	55	29	81	7,10-13,17,24-33,39,41,47,48
DBPs	89	Bromoform	15	10	20	18
	90	Chloroform	2800	n/a	n/a	18
	91	Nitrosodimethylamine	102	70	130	18
	92	Nitrosopiperidine	160	n/a	n/a	18
	93	Nitrosopyrrolidine	225	180	270	18

[1]A triangular distribution was utilized to estimate relevant uncertainties where median, minimum, and maximum values were available. Conversely, a uniform distribution was applied in cases where only point values were provided. The unit is ng L^{-1}.

Supplementary Table 10 | Assumptions for the removal efficiencies of CECs in the RBF-RO and RBF-ET systems[1].

System	No	Process	Pesticides				Pharmaceuticals				Other categories			
			Median	Min	Max	Reference	Median	Min	Max	Reference	Median	Min	Max	Reference
RBF-RO	1	Riverbank filtration	50%	36%	63%	[50-53]	74%	68%	81%	[41,50-59]	64%	56%	71%	[41,50-59]
	2	Reverse osmosis	61%	72%	78%	[60]	93.6%	94.6%	95.3%	[60-64]	85%	87%	90%	[60-65]
	3	Ion exchange	n/a	n/a	n/a		n/a	n/a	n/a		n/a	n/a	n/a	
	4	Remineralization	n/a	n/a	n/a		n/a	n/a	n/a		n/a	n/a	n/a	
	5	Aeration and degasification	n/a	n/a	n/a		n/a	n/a	n/a		n/a	n/a	n/a	
	6	Ultraviolet	n/a	n/a	n/a		n/a	n/a	n/a		n/a	n/a	n/a	
RBF-ET	1	Riverbank filtration	50%	36%	63%	[50-53]	74%	68%	81%	[41,50-59]	64%	56%	71%	[41,50-59]
	2	Biological iron removal	n/a	n/a	n/a		n/a	n/a	n/a		n/a	n/a	n/a	
	3	Pellet softening	n/a	n/a	n/a		n/a	n/a	n/a		n/a	n/a	n/a	
	4	Carry-over filter	27%	23%	31%	[40,66-68]	27%	23%	31%	[40,66-68]	27%	23%	31%	[40,66-68]
	5	Ion exchange	n/a	n/a	n/a		n/a	n/a	n/a		n/a	n/a	n/a	
	6	Granular activated sludge	61%	48%	72%	[19,68]	67%	58%	73%	[19,67-70]	66%	60%	72%	[19,67-70]
	7	Ultraviolet	n/a	n/a	n/a		n/a	n/a	n/a		n/a	n/a	n/a	

[1]A triangular distribution was used to estimate the relevant uncertainties.

Supplementary Table 11 | Modeled concentrations of CECs in the RBF-RO and RBF-ET products[1].

Category	No	Chemicals	RBF-RO water			RBF-ET water		
			Median	Min	Max	Median	Min	Max
Pesticides	1	1,4-Dichlorobenzene	22.81	12.91	35.78	22.53	12.81	36.99
	2	α-HCH	0.006	0.003	0.01	0.006	0.003	0.009
	3	Atrazine	5.84	3.12	11.17	0.14	0.09	0.23
	4	Azinphos-methyl	1.17	0.65	2.08	1.16	0.57	1.96
	5	Bentazone	3.40	1.02	7.35	1.82	0.52	3.99
	6	Buprofezin	0.63	0.31	1.25	0.62	0.27	1.35
	7	Carbaryl	3.68	2.12	6.01	3.64	2.01	6.09
	8	Carbendazim	15.59	3.08	39.41	4.47	0.86	10.10
	9	Carbofuran	3.85	0.53	11.83	4.24	0.37	18.90
	10	Chlorfenvinphos	1.19	0.22	3.36	1.19	0.24	3.33
	11	Chlorpyrifos	0.91	0.22	2.35	0.90	0.23	2.26
	12	Diazinon	4.08	1.38	9.65	1.26	0.30	3.37
	13	Diflubenzuron	0.88	0.53	1.40	0.87	0.48	1.45
	14	Dimethoate	4.26	0.78	11.71	2.95	0.52	7.59
	15	Diuron	0.91	0.21	3.53	2.53	0.30	10.52
	16	Ethion	3.90	1.50	8.31	3.89	1.27	8.15
	17	Fenitrothion	6.02	3.34	10.52	5.98	2.80	10.89
	18	Fipronil	2.94	0.29	9.67	5.18	0.47	18.87
	19	Hexachlorobenzene	0.006	0.003	0.01	0.006	0.003	0.009
	20	Hexythiazox	1.45	0.32	3.40	1.43	0.33	3.70
	21	Imazalil	8.47	0.71	24.23	8.42	0.71	24.68
	22	Imidacloprid	2.86	0.54	6.49	2.08	0.35	4.20
	23	Iprodione	0.29	0.17	0.47	0.29	0.16	0.47
	24	Linuron	0.44	0.25	0.74	0.44	0.24	0.72
	25	Malathion	0.63	0.29	1.27	0.62	0.30	1.29
	26	MCPA	81.14	52.64	122.14	64.93	37.61	109.43
	27	Mecoprop	9.82	4.93	17.47	31.21	16.45	54.56
	28	Methamidophos	69.58	7.07	184.53	68.40	6.03	183.57
	29	Methiocarb	0.43	0.24	0.73	0.42	0.22	0.76
	30	Metoalachlor	8.09	1.61	22.70	8.01	1.44	23.71
	31	p,p'-DDD	0.001	0.0008	0.002	0.0015	0.0008	0.0024

	32	p,p'-DDE	0.0006	0.0003	0.0009	0.0006	0.0003	0.0009
	33	Prochloraz	1.96	0.59	4.54	1.95	0.65	4.52
	34	Promethryn	0.02	0.00	0.05	0.01	0.00	0.04
	35	Propazine	0.96	0.49	1.94	0.96	0.49	1.98
	36	Propiconazole	0.15	0.08	0.24	0.03	0.00	0.09
	37	Pyriproxyphen	1.61	0.12	4.45	1.60	0.16	4.16
	38	Simazine	2.68	1.21	5.29	2.64	1.16	5.21
	39	Tebuconazole	1.63	0.28	3.68	1.21	0.19	3.22
	40	Terbutryn	1.40	0.26	3.94	1.36	0.28	3.76
	41	Thiabendazole	2.33	0.35	6.88	2.32	0.33	6.54
Industrial chemicals	42	1H–Benzotriazole	16.98	9.21	29.03	33.40	18.15	57.20
	43	Acetophenone	4.11	2.04	7.39	8.07	4.17	14.81
	44	Anthracene	0.14	0.10	0.19	0.27	0.19	0.38
	45	Butylbenzyl phthalate	2.44	1.71	3.40	4.81	3.29	6.69
	46	Carbazole	0.46	0.32	0.63	0.91	0.63	1.26
	47	Dibutyl phthalate	334.96	31.84	814.85	661.42	67.47	1601.15
	48	Diethyl phthalate	37.92	11.59	77.35	75.08	20.97	156.66
	49	Diethylhexyl phthalate	6.94	4.89	9.66	13.63	9.39	18.49
	50	Fluoranthene	0.09	0.06	0.13	0.18	0.13	0.26
	51	Fluorene	0.23	0.16	0.32	0.45	0.31	0.63
	52	Isophorone	0.09	0.06	0.13	0.18	0.13	0.25
	53	Naphthalene	78.98	0.11	322.89	152.48	0.28	665.94
	54	Nitrobenzene	7.99	0.03	30.56	15.92	0.10	63.65
	55	Phenanthrene	0.32	0.22	0.45	0.63	0.43	0.88
	56	Phenol	2.35	1.65	3.25	4.62	3.20	6.36
	57	Quinoline	0.32	0.23	0.44	0.64	0.44	0.87
	58	TCEP	3.56	1.34	6.71	14.42	5.66	26.90
	59	Tributyl phosphate	7.27	4.98	10.18	14.32	9.84	20.00
	60	Tris-1,3-dichloro-2-propyl phosphate	13.21	3.14	29.97	25.93	5.33	55.43
Pharmaceuticals	61	Acetaminophen	60.80	1.92	170.96	215.46	4.09	1004.60
	62	Antipyrine	0.13	0.05	0.25	0.20	0.08	0.37
	63	Bezafibrate	0.96	0.02	6.98	2.55	0.17	11.15
	64	Carbamazepine	6.39	2.21	11.30	10.17	2.59	20.84
	65	Diclofenac	5.72	2.77	10.08	47.60	18.17	99.60
	66	Ethinylestradiol	0.05	0.03	0.06	0.09	0.06	0.12

	67	Furosemide	3.00	0.79	7.60	5.86	1.66	14.29
	68	Gabapentin	1.85	0.03	7.98	12.00	0.12	49.51
	69	Gemfibrozil	0.08	0.03	0.17	1.15	0.36	2.92
	70	Indomethacin	1.88	0.08	6.15	2.02	0.12	6.53
	71	Iopromide	3.30	0.20	10.73	7.83	0.42	25.09
	72	Metoprolol	0.16	0.02	0.44	2.28	0.11	12.27
	73	Oxazepam	0.35	0.21	0.55	0.74	0.24	2.11
	74	Phenytoin	0.52	0.34	0.76	1.01	0.60	1.55
	75	Primidone	0.36	0.24	0.52	0.56	0.33	0.93
	76	Triamterene	1.01	0.65	1.45	1.97	1.18	2.99
	77	Zidovudine	8.68	0.11	25.12	13.47	0.19	37.55
PCPs	78	Butylated hydroxy toluene	2.26	1.60	3.09	4.43	3.07	6.19
	79	Butylated hydroxyanisole	21.91	14.90	30.29	43.11	29.67	60.20
	80	Chlorophene	0.33	0.12	0.67	0.65	0.25	1.38
	81	Musk xylene	0.04	0.03	0.04	0.91	0.63	1.27
Antibiotics	82	Dimetridazole	0.42	0.30	0.57	0.82	0.58	1.13
	83	Metronidazole	0.24	0.11	0.44	0.48	0.23	0.92
	84	Nalidixic acid	1.25	0.23	3.51	2.50	0.48	7.10
	85	Oxolinic acid	1.83	0.80	3.40	3.62	1.54	6.72
	86	Sulfadimidine	0.30	0.11	0.64	3.14	1.23	6.26
	87	Sulfasalazine	1.60	0.67	3.13	3.14	1.23	6.26
	88	Trimethoprim	0.26	0.03	0.74	0.38	0.04	1.16
DBPs	89	Bromoform	0.69	0.38	1.23	1.36	0.71	2.31
	90	Chloroform	129.23	92.63	177.20	254.27	177.74	348.45
	91	Nitrosodimethylamine	4.62	2.55	7.60	9.13	4.69	16.38
	92	Nitrosopiperidine	10.38	6.38	15.87	14.49	9.70	20.52
	93	Nitrosopyrrolidine	7.39	5.25	10.11	20.33	12.64	32.60

[1]The unit is ng L^{-1}.

Supplementary Table 12 | Life cycle inventories of the RBF-RO and RBF-ET systems.				
System	Process	Inventory	Value	Unit
RBF-RO	Riverbank filtration	Electricity	2.38×10^{-1}	kWh m^{-3}
		Water	1.25	m^3 m^{-3}
		H_2O_2	5.00×10^{-4}	L m^{-3}
	Reverse osmosis	Electricity	5.00×10^{-1}	kWh m^{-3}
		Antiscalant	2.60×10^{-6}	ton m^{-3}
		NaOH	5.00×10^{-4}	L m^{-3}
		Sodium sulfite	2.00×10^{-3}	L m^{-3}
		Membrane	4.17×10^{-7}	ton m^{-3}
		Wastewater	2.50×10^{-1}	m^3 m^{-3}
	Ion exchange	Electricity	4.75×10^{-2}	kWh m^{-3}
		Resin	9.58×10^{-7}	ton m^{-3}
		HCl	6.08×10^{-6}	ton m^{-3}
		Wastewater	6.79×10^{-4}	m^3 m^{-3}
	Remineralization	Electricity	3.33×10^{-3}	kWh m^{-3}
		Granular calcite	1.00×10^{-4}	ton m^{-3}
		CO_2	2.64×10^{-4}	ton m^{-3}
	Aeration and degasification	Electricity	1.30×10^{-1}	kWh m^{-3}
		Emission of CO_2	2.00×10^{-6}	ton m^{-3}
		Emission of CH_4	1.32×10^{-4}	ton m^{-3}
	Ultraviolet	Electricity	5.83×10^{-2}	kWh m^{-3}
		UV lamps	1.33×10^{-5}	kg m^{-3}
RBF-ET	Riverbank filtration	Electricity	2.00×10^{-1}	kWh m^{-3}
		Water	1.04	m3 m^{-3}
		H_2O_2	5.00×10^{-4}	L m^{-3}
	Biological iron removal	Electricity	1.37×10^{-1}	kWh m^{-3}
		Sand	1.83×10^{-5}	ton m^{-3}
		Emission of CO_2	2.00×10^{-6}	ton m^{-3}
	Pellet softening	NaOH	2.00×10^{-4}	ton m^{-3}
		H_2SO_4	2.00×10^{-4}	ton m^{-3}
		Sand	3.46×10^{-5}	ton m^{-3}
	Carry-over filter	Electricity	1.96×10^{-2}	kWh m^{-3}
		Sand	1.83×10^{-5}	ton m^{-3}
		Polyelectrolyte	8.75×10^{-9}	ton m^{-3}
		sludge	1.88×10^{-5}	ton m^{-3}
	Ion exchange	Electricity	3.25×10^{-5}	kWh m^{-3}
		Resin	9.58×10^{-7}	ton m^{-3}
		HCl	6.08×10^{-6}	ton m^{-3}
		Wastewater	6.79×10^{-4}	m^3 m^{-3}
	Granular activated carbon	Activated carbon	2.08×10^{-6}	ton m^{-3}
		Activated carbon regeneration	1.08×10^{-5}	ton m^{-3}
	Ultraviolet	Electricity	5.83×10^{-2}	kWh m^{-3}
		Lamps	1.33×10^{-5}	kg m^{-3}
Wastewater treatment		Electricity	3.28×10^{-1}	kWh m^{-3}
		Recovered electricity	1.01×10^{-1}	kWh m^{-3}
		Polyelectrolyte	1.67×10^{-8}	ton m^{-3}
		Sludge	3.17×10^{-5}	ton m^{-3}

Sludge disposal	Electricity	1.25	kWh ton^{-1}
	Renewed electricity	5.00×10^{-4}	kWh ton^{-1}
	Emission of CO_2	5.00×10^{-1}	kg ton^{-1}
	Emission of N_2O	2.60×10^{-6}	g ton^{-1}

Supplementary Table 13| Inventories of transportation of consumables in the RBF-RO and RBF-ET systems.

System	Process	Inventory	Weight (ton m^{-3})	Distance (km)
RBF-RO	Riverbank filtration	H_2O_2	5.46×10^{-7}	90
	Reverse osmosis	Antiscalant	2.60×10^{-6}	450
		Critic acid	8.33×10^{-7}	90
		NaOH	1.07×10^{-6}	180
		Sodium sulfite	5.27×10^{-6}	90
		Membrane	1.04×10^{-5}	30000
		Sludge to incinerator	7.92×10^{-6}	50
		Waste membrane to incinerator	1.04×10^{-5}	50
	Ion exchange	Resin	2.40×10^{-5}	700
		Sludge to incinerator	2.51×10^{-8}	50
	Remineralization	Granular calcite	1.00×10^{-4}	500
		CO_2	3.57×10^{-4}	150
	Ultraviolet	Lamp	3.33×10^{-7}	100
RBF-ET	Riverbank filtration	H_2O_2	5.46×10^{-7}	90
	Biological iron removal	Sand	9.17×10^{-4}	80
	Pellet softening	NaOH	2.00×10^{-4}	180
	Pellet softening	H_2SO_4	2.00×10^{-4}	90
	Carry-over filter	Sand	3.46×10^{-5}	80
		Sludge to incinerator	1.88×10^{-5}	50
	Ion exchange	Resin	2.40×10^{-5}	700
		Sludge to incinerator	2.15×10^{-8}	50
	Granular activated carbon	Activated carbon	5.21×10^{-5}	700
		Activated carbon regeneration	2.71×10^{-4}	600
	Ultraviolet	Lamp	3.33×10^{-7}	100

The above-mentioned parameters and values are summarized from literature[2].

Supplementary Table 14 | Selected inventory processes in the Ecoinvent database.	
**Item**	**Unit process**
H_2O_2	Hydrogen peroxide, without water, in 50% solution state {RER}| hydrogen peroxide production, product in 50% solution state | APOS, U
Antiscalant	Polycarboxylates, 40% active substance {RER}| production | APOS, U
Citric acid	Citric acid {RER}| production | APOS, U
NaOH	Sodium hydroxide, without water, in 50% solution state {RER}| chlor-alkali electrolysis, membrane cell | APOS, U
Sodium sulfite	Sodium sulfite {RER}| production | APOS, U
Membrane	Glass fibre reinforced plastic, polyamide, injection moulded {RER}| production | APOS, U, Extrusion, plastic film {RER}| production | APOS, U
Resin	Cationic resin {CH}| production | APOS, U
HCl	Hydrochloric acid, without water, in 30% solution state {RER}| allyl chloride production, reaction of propylene and chlorine | APOS, U
Granular calcite	Calcium carbonate > 63 microns, production, at plant EU-27 S
CO_2	Carbon dioxide, liquid {RER}| production | APOS, U
UV lamps	Ultraviolet lamp| ultraviolet lamp production, for water disinfection | APOS, U
Energy	Electricity, high voltage {NL}| market for electricity, high voltage | APOS, U
Waste Membrane disposal	Waste incineration of plastics (PE, PP, PS, PB), EU-27
Polyelectrolyte	Polyacrylamide| production | APOS, U
Membrane Transport	Transport, freight, sea, transoceanic ship| market for | APOS, U; Transport, freight, sea, transoceanic ship| market for | APOS, U
Transport	Transport, freight, lorry 16-32 metric ton, EURO5| market for | APOS, U;
Sand	Silica sand {DE}| production | APOS, U
H_2SO_4	Sulfuric acid {RER}| production | APOS, U
Activated carbon	Activated carbon, granular {RER}| activated carbon production, granular from hard coal | APOS, U
Activated carbon regeneration	Activated carbon, granular {RER}| treatment of spent activated carbon, granular from hard coal, reactivation | APOS, U

Supplementary Table 15 | Overview of the selected environmental impact categories used in this analysis.

Impact category	Unit	Description
Global warming	kg CO_2 eq	The global warming estimated feedback of the GHGs emission in atmosphere. It is expressed by the global warming potency of CO_2 equivalents.
Ozone depletion	kg CFC11 eq	Stratospheric ozone depletion expressed the reduction of ozone level in atmosphere due to the anthropogenic emissions. The ozone depletion of a chemical is equal to that of CFC11.
Terrestrial acidification	kg SO_2 eq	The terrestrial acidification potential was based on the dry or wet decomposition of acidic gases. The acidification potency is measured by equivalent acidity of SO_2.
Freshwater eutrophication	kg P eq	The freshwater eutrophication potential derived from of environment fate of nutrient emission. It is estimated by equivalent eutrophication potential of P containing chemicals.
Marine eutrophication	kg N eq	The potency of marine eutrophication caused by the excess nutrients in water body. Marine eutrophication is characterized by that of equivalent substance in terms of N.
Terrestrial ecotoxicity	kg 1,4-DCB	Terrestrial ecotoxicity estimated the harmful effect on soil during the transformation of substance. The potential of contamination is equal to that of 1,4-DCB.
Freshwater ecotoxicity	kg 1,4-DCB	Freshwater ecotoxicity describe the toxicity of substance to freshwater ecosystem. It is expressed by the equivalent freshwater eco-toxicity effect of 1,4-DCB.
Marine ecotoxicity	kg 1,4-DCB	Marine ecotoxicity potential was based on the potency of causing toxicity on marine compartment. The unit is kg 1,4-DCB equivalent.
Mineral consumption	kg Cu eq	Mineral consumption expressed the depletion of mineral resource due to the investment. It is estimated by the equivalent mineral resource scarcity of Cu consumption.
Fossil resource depletion	kg oil eq	The fossil fuel depletion described implication of fossil fuel energy use. It is characterized by the surplus ore potential.

Supplementary Table 16 | Country-specific processes for electricity generation in the Ecoinvent database.

Country	Continent	Region	Unit process
Eritrea	Africa	Eastern	Electricity, high voltage {ER}| market for electricity, high voltage | APOS, U
Ethiopia	Africa	Eastern	Electricity, high voltage {ET}| market for electricity, high voltage | APOS, U
Kenya	Africa	Eastern	Electricity, high voltage {KE}| market for electricity, high voltage | APOS, U
Mauritius	Africa	Eastern	Electricity, high voltage {MU}| market for electricity, high voltage | APOS, U
Mozambique	Africa	Eastern	Electricity, high voltage {MZ}| market for electricity, high voltage | APOS, U
South Sudan	Africa	Eastern	Electricity, high voltage {SS}| market for electricity, high voltage | APOS, U
United Republic of Tanzania	Africa	Eastern	Electricity, high voltage {TZ}| market for | APOS, U
Zambia	Africa	Eastern	Electricity, high voltage {ZM}| market for electricity, high voltage | APOS, U
Zimbabwe	Africa	Eastern	Electricity, high voltage [71]| market for electricity, high voltage | APOS, U
Congo	Africa	Middle	Electricity, high voltage [71]| market for electricity, high voltage | APOS, U
Angola	Africa	Middle	Electricity, high voltage {AO}| market for electricity, high voltage | APOS, U
Congo, Democratic Republic of the	Africa	Middle	Electricity, high voltage {CD}| market for electricity, high voltage | APOS, U
Cameroon	Africa	Middle	Electricity, high voltage [71]| market for electricity, high voltage | APOS, U
Gabon	Africa	Middle	Electricity, high voltage {GA}| market for electricity, high voltage | APOS, U
Egypt	Africa	Northern	Electricity, high voltage {EG}| market for electricity, high voltage | APOS, U
Algeria	Africa	Northern	Electricity, high voltage {DZ}| market for electricity, high voltage | APOS, U
Libya	Africa	Northern	Electricity, high voltage {LY}| market for electricity, high voltage | APOS, U
Morocco	Africa	Northern	Electricity, high voltage {MA}| market for electricity, high voltage | APOS, U
Sudan	Africa	Northern	Electricity, high voltage {SD}| market for electricity, high voltage | APOS, U
Tunisia	Africa	Northern	Electricity, high voltage {TN}| market for electricity, high voltage | APOS, U
South Africa	Africa	Southern	Electricity, high voltage {ZA}| market for | APOS, U

Botswana	Africa	Southern	Electricity, high voltage {BW}| market for electricity, high voltage | APOS, U
Namibia	Africa	Southern	Electricity, high voltage {NA}| market for electricity, high voltage | APOS, U
Benin	Africa	Western	Electricity, high voltage {BJ}| market for electricity, high voltage | APOS, U
Cote d'Ivoire	Africa	Western	Electricity, high voltage {CI}| market for electricity, high voltage | APOS, U
Ghana	Africa	Western	Electricity, high voltage {GH}| market for electricity, high voltage | APOS, U
Niger	Africa	Western	Electricity, high voltage {NE}| market for electricity, high voltage | APOS, U
Nigeria	Africa	Western	Electricity, high voltage {NG}| market for electricity, high voltage | APOS, U
Senegal	Africa	Western	Electricity, high voltage {SN}| market for electricity, high voltage | APOS, U
Togo	Africa	Western	Electricity, high voltage {TG}| market for electricity, high voltage | APOS, U
Cuba	Americas	Caribbean	Electricity, high voltage {CU}| market for electricity, high voltage | APOS, U
Dominican Republic	Americas	Caribbean	Electricity, high voltage {DO}| market for electricity, high voltage | APOS, U
Haiti	Americas	Caribbean	Electricity, high voltage {HT}| market for electricity, high voltage | APOS, U
Jamaica	Americas	Caribbean	Electricity, high voltage {JM}| market for electricity, high voltage | APOS, U
Trinidad and Tobago	Americas	Caribbean	Electricity, high voltage {TT}| market for electricity, high voltage | APOS, U
Costa Rica	Americas	Central	Electricity, high voltage {CR}| market for electricity, high voltage | APOS, U
Guatemala	Americas	Central	Electricity, high voltage {GT}| market for electricity, high voltage | APOS, U
Honduras	Americas	Central	Electricity, high voltage {HN}| market for electricity, high voltage | APOS, U
Mexico	Americas	Central	Electricity, high voltage {MX}| market for | APOS, U
Nicaragua	Americas	Central	Electricity, high voltage {NI}| market for electricity, high voltage | APOS, U
Panama	Americas	Central	Electricity, high voltage {PA}| market for electricity, high voltage | APOS, U
El Salvador	Americas	Central	Electricity, high voltage {SV}| market for electricity, high voltage | APOS, U
Canada	Americas	North	Electricity, high voltage {CA}| market group for | APOS, U

United States	Americas	North	Electricity, high voltage {US}| market group for | APOS, U
Argentina	Americas	South	Electricity, high voltage {AR}| market for electricity, high voltage | APOS, U
Brazil	Americas	South	Electricity, high voltage {BR}| market for | APOS, U
Plurinational State of Bolivia	Americas	South	Electricity, high voltage {BO}| market for electricity, high voltage | APOS, U
Chile	Americas	South	Electricity, high voltage {CL}| market for | APOS, U
Colombia	Americas	South	Electricity, high voltage {CO}| market for electricity, high voltage | APOS, U
Ecuador	Americas	South	Electricity, high voltage {EC}| market for electricity, high voltage | APOS, U
Peru	Americas	South	Electricity, high voltage {PE}| market for | APOS, U
Paraguay	Americas	South	Electricity, high voltage {PY}| market for electricity, high voltage | APOS, U
Uruguay	Americas	South	Electricity, high voltage {UY}| market for electricity, high voltage | APOS, U
Venezuela	Americas	South	Electricity, high voltage {VE}| market for electricity, high voltage | APOS, U
China	Asia	Eastern	Electricity, high voltage {CN}| market group for | APOS, U
Republic of Korea	Asia	Eastern	Electricity, high voltage {KR}| market for | APOS, U
Japan	Asia	Eastern	Electricity, high voltage {JP}| market for | APOS, U
Democratic People's Republic of Korea	Asia	Eastern	Electricity, high voltage [71]| market for electricity, high voltage | APOS, U
Mongolia	Asia	Eastern	Electricity, high voltage {MN}| market for electricity, high voltage | APOS, U
India	Asia	South-central	Electricity, high voltage {IN}| market group for electricity, high voltage | APOS, U
Bangladesh	Asia	South-central	Electricity, high voltage {BD}| market for electricity, high voltage | APOS, U
Brunei Darussalam	Asia	South-central	Electricity, high voltage [72]| market for electricity, high voltage | APOS, U
Iran (Islamic Republic of)	Asia	South-central	Electricity, high voltage {IR}| market for | APOS, U
Kyrgyzstan	Asia	South-central	Electricity, high voltage {KG}| market for electricity, high voltage | APOS, U
Kazakhstan	Asia	South-central	Electricity, high voltage {KZ}| market for electricity, high voltage | APOS, U
Sri Lanka	Asia	South-central	Electricity, high voltage {LK}| market for electricity, high voltage | APOS, U
Nepal	Asia	South-central	Electricity, high voltage {NP}| market for electricity, high voltage | APOS, U

Pakistan	Asia	South-central	Electricity, high voltage {PK}| market for electricity, high voltage | APOS, U
Tajikistan	Asia	South-central	Electricity, high voltage {TJ}| market for electricity, high voltage | APOS, U
Turkmenistan	Asia	South-central	Electricity, high voltage {TM}| market for electricity, high voltage | APOS, U
Uzbekistan	Asia	South-central	Electricity, high voltage {UZ}| market for electricity, high voltage | APOS, U
Singapore	Asia	South-eastern	Electricity, high voltage [71]| market for electricity, high voltage | APOS, U
Vietnam	Asia	South-eastern	Electricity, high voltage [71]| market for electricity, high voltage | APOS, U
Indonesia	Asia	South-eastern	Electricity, high voltage {ID}| market for | APOS, U
Cambodia	Asia	South-eastern	Electricity, high voltage {KH}| market for electricity, high voltage | APOS, U
Myanmar	Asia	South-eastern	Electricity, high voltage {MM}| market for electricity, high voltage | APOS, U
Malaysia	Asia	South-eastern	Electricity, high voltage {MY}| market for | APOS, U
Philippines	Asia	South-eastern	Electricity, high voltage {PH}| market for electricity, high voltage | APOS, U
Thailand	Asia	South-eastern	Electricity, high voltage {TH}| market for | APOS, U
Turkey	Asia	Western	Electricity, high voltage {TR}| market for | APOS, U
United Arab Emirates	Asia	Western	Electricity, high voltage {AE}| market for electricity, high voltage | APOS, U
Armenia	Asia	Western	Electricity, high voltage {AM}| market for electricity, high voltage | APOS, U
Azerbaijan	Asia	Western	Electricity, high voltage {AZ}| market for electricity, high voltage | APOS, U
Bahrain	Asia	Western	Electricity, high voltage {BH}| market for electricity, high voltage | APOS, U
Cyprus	Asia	Western	Electricity, high voltage {CY}| market for | APOS, U
Georgia	Asia	Western	Electricity, high voltage {GE}| market for electricity, high voltage | APOS, U
Israel	Asia	Western	Electricity, high voltage {IL}| market for electricity, high voltage | APOS, U
Iraq	Asia	Western	Electricity, high voltage {IQ}| market for electricity, high voltage | APOS, U
Jordan	Asia	Western	Electricity, high voltage {JO}| market for electricity, high voltage | APOS, U
Kuwait	Asia	Western	Electricity, high voltage {KW}| market for electricity, high voltage | APOS, U

Lebanon	Asia	Western	Electricity, high voltage {LB}| market for electricity, high voltage | APOS, U
Qatar	Asia	Western	Electricity, high voltage {QA}| market for electricity, high voltage | APOS, U
Saudi Arabia	Asia	Western	Electricity, high voltage {SA}| market for | APOS, U
Syrian Arab Republic	Asia	Western	Electricity, high voltage {SY}| market for electricity, high voltage | APOS, U
Yemen	Asia	Western	Electricity, high voltage {YE}| market for electricity, high voltage | APOS, U
the Czech Republic	Europe	Eastern	Electricity, high voltage {CZ}| market for | APOS, U
Belarus	Europe	Eastern	Electricity, high voltage {BY}| market for electricity, high voltage | APOS, U
Hungary	Europe	Eastern	Electricity, high voltage {HU}| market for | APOS, U
Republic of Moldova	Europe	Eastern	Electricity, high voltage {MD}| market for electricity, high voltage | APOS, U
Poland	Europe	Eastern	Electricity, high voltage {PL}| market for | APOS, U
Romania	Europe	Eastern	Electricity, high voltage {RO}| market for | APOS, U
Russia	Europe	Eastern	Electricity, high voltage {RU}| market for | APOS, U
Slovakia	Europe	Eastern	Electricity, high voltage {SK}| market for | APOS, U
Ukraine	Europe	Eastern	Electricity, high voltage {UA}| market for | APOS, U
Denmark	Europe	Northern	Electricity, high voltage {DK}| market for | APOS, U
Estonia	Europe	Northern	Electricity, high voltage {EE}| market for | APOS, U
Finland	Europe	Northern	Electricity, high voltage {FI}| market for | APOS, U
United Kingdom	Europe	Northern	Electricity, high voltage {GB}| market for | APOS, U
Ireland	Europe	Northern	Electricity, high voltage {IE}| market for | APOS, U
Iceland	Europe	Northern	Electricity, high voltage {IS}| market for | APOS, U
Lithuania	Europe	Northern	Electricity, high voltage {LT}| market for | APOS, U
Latvia	Europe	Northern	Electricity, high voltage {LV}| market for | APOS, U
Norway	Europe	Northern	Electricity, high voltage {NO}| market for | APOS, U
Sweden	Europe	Northern	Electricity, high voltage {SE}| market for | APOS, U

Albania	Europe	Southern	Electricity, high voltage {AL}| market for electricity, high voltage | APOS, U
Bosnia and Herzegovina	Europe	Southern	Electricity, high voltage {BA}| market for | APOS, U
Spain	Europe	Southern	Electricity, high voltage {ES}| market for | APOS, U
Greece	Europe	Southern	Electricity, high voltage {GR}| market for | APOS, U
Croatia	Europe	Southern	Electricity, high voltage {HR}| market for | APOS, U
Italy	Europe	Southern	Electricity, high voltage {IT}| market for | APOS, U
Montenegro	Europe	Southern	Electricity, high voltage {ME}| market for electricity, high voltage | APOS, U
North Macedonia	Europe	Southern	Electricity, high voltage {MK}| market for | APOS, U
Malta	Europe	Southern	Electricity, high voltage {MT}| market for | APOS, U
Portugal	Europe	Southern	Electricity, high voltage {PT}| market for | APOS, U
Serbia	Europe	Southern	Electricity, high voltage {RS}| market for | APOS, U
Slovenia	Europe	Southern	Electricity, high voltage {SI}| market for | APOS, U
Netherlands	Europe	Western	Electricity, high voltage {NL}| market for | APOS, U
Austria	Europe	Western	Electricity, high voltage {AT}| market for | APOS, U
Belgium	Europe	Western	Electricity, high voltage {BE}| market for | APOS, U
Bulgaria	Europe	Western	Electricity, high voltage {BG}| market for | APOS, U
Switzerland	Europe	Western	Electricity, high voltage {CH}| market for | APOS, U
Germany	Europe	Western	Electricity, high voltage {DE}| market for | APOS, U
France	Europe	Western	Electricity, high voltage {FR}| market for | APOS, U
Luxembourg	Europe	Western	Electricity, high voltage {LU}| market for | APOS, U
New Zealand	Oceania	Australias	Electricity, high voltage {NZ}| market for electricity, high voltage | APOS, U
Australia	Oceania	Australias	Electricity, high voltage {AU}| market for | APOS, U

Supplementary Table 17 | List of CECs and process configurations considered in Supplementary Fig. 5.			
Category	Chemical (CAS number)	Configuration	Source
Pesticides	Acetamiprid (135410-20-7), Atrazine (1912-24-9), Azoxystrobin (131860-33-8), Bentazon (25057-89-0), Buprofezin (69327-76-0), Chlorantraniliprole (500008-45-7), Clorpirifos (2921-88-2), Clothianidin (210880-92-5), Desethyl-Atrazine (6190-65-4), Dimethachlor (50563-36-5), Diuron (330-54-1), Imidacloprid (138261-41-3), Isoproturon (34123-59-6), Lindane (58-89-9), Metolachlor (51218-45-2), p´p-DDD (72-54-8), Propiconazole (60207-90-1), Pymetrozine (123312-89-0), Simazine (122-34-9), Tebuconazole (107534-96-3), Terbuthylazine (5915-41-3), Terbutryn (886-50-0), Thiacloprid (111988-49-9), Thiamethoxam (153719-23-4), Tricyclazole (41814-78-2), Trifloxystrobin (141517-21-7), γ-Chlordane (5103-74-2)	Water treatment: i) traditional water treatment (including coagulation, flocculation-sedimentation, filtration and chlorination); ii) Integration of traditional water treatment, ozonation and granular activated carbon; iii) integration of pre-ozonation and traditional water treatment, ozonation and granular activated carbon	[73-79]
Industrial chemicals	5,6-dimethylbenzotriazole (4184-79-6), 5-chlorobenzotriazole (94-97-3), 5-methyl-benzotriazole (136-85-6), 6:2 fluorotelomer sulfonic acid (27619-97-2), Benzotriazole (95-14-7), Bisphenol A (80-05-7), Ethylhexyldiphenylphosphate (1241-94-7), Nonylphenol (84852-15-3), Perfluorobenzenesulfonic acid (30334-69-1), Perfluorobutane sulfonic acid (375-73-5), Perfluorobutanoate (375-22-4), Perfluorodecanoic acid (335-76-2), Perfluoroheptane sulfonate (375-92-8), Perfluoroheptanoic acid (375-85-9), Perfluorohexane sulfonic acid (355-46-4), Perfluorohexanoic acid (307-24-4), Perfluorononanoic acid (375-95-1), Perfluorooctane sulfonic acid (1763-23-1), Perfluorooctanoic acid (335-67-1), Perfluoropentanoic acid (2706-90-3), Tri-isobutyl phosphate (126-71-6), Tri-n-butyl phosphate (126-73-8), Tris-(2-chloro-, 1-chloromethyl-ethyl) phosphate (13674-87-8), Tris-(2-chloro-, 1-methyl-ethyl) phosphate (13674-84-5), Tris-(2-chloroethyl) phosphate (115-96-8), Tris-(butoxyethyl) phosphate (78-51-3)	I. Water treatment: i) biologically active slow sand filters, underground passage and soil passage; ii) integration of traditional water treatment and granular activated carbon; iii) integration of traditional water treatment, ozonation and granular activated carbon; iv) integration of pre-ozonation and traditional water treatment, ozonation and granular activated carbon. II. Wastewater treatment: i) traditional activated sludge; ii) membrane bioreactor; iii) sequencing batch reactor or cyclic activated sludge system; iv) traditional water treatment waste stabilization, lagoons-dissolved air flotation/filtration; v) UNITANK.	[73,77,80-84]
Pharmaceuticals	Acetaminophen (103-90-2), Amitriptyline (50-48-6), Antipyrine (60-80-0), Atenolol (29122-	I. Water treatment: i) traditional water	[69,73,77,78,81,82,85-87]

	68-7), Benzoylecgonine (519-09-5), Bisoprolol (66722-44-9), Caffeine (58-08-2), Carbamazepine (298-46-4), Citalopram (59729-33-8), Cotinine (486-56-6), Crotamiton (483-63-6), Diazepam (439-14-5), Diclofenac (15307-86-5), Fluoxetine (54910-89-3), Furosemide (54-31-9), Gabapentin (60142-96-3), Gemfibrozil (25812-30-0), Ibuprofen (15687-27-1), Iohexol (66108-95-0), Iopamidol (60166-93-0), Iopromide (73334-07-3), Irbesartan (138402-11-6), Levofloxacin (100986-85-4), Metoprolol (51384-51-1), Naproxen (22204-53-1), Nicotine (22083-74-5), Paroxetine (61869-08-7), Salicylic Acid (69-72-7), Sertraline (79617-96-2), Sotalol (3930-20-9), Sulfamethazine (35762-76-6), Sulpiride (15676-16-1), Trimethoprim (738-70-5), Valsartan (137862-53-4), Venlafaxine (93413-69-5)	treatment (including coagulation, flocculation-sedimentation, filtration and chlorination); ii) integration of pre-ozonation and traditional water treatment; iii) integration of traditional water treatment and granular activated carbon; iv) integration of traditional water treatment and membrane filtration; v) integration of traditional water treatment and ozonation, granular activated carbon; vi) integration of pre-ozonation and traditional water treatment, ozonation and granular activated carbon.<br>II. Wastewater treatment: i) traditional activated sludge; ii) sequencing batch reactor or cyclic activated sludge system; iii) membrane bioreactor; iv) traditional activated sludge with extended aeration.	
Antibiotics	Clarithromycin (81103-11-9), Doxycycline (564-25-0), Erythromycin (114-07-8), Lincomycin (154-21-2), Oxytetracycline (79-57-2), Roxithromycin (80214-83-1), Sulfadiazine (68-35-9), Sulfamethoxazole (723-46-6), Sulfamonomethoxine (1220-83-3)	I. Water treatment: i) integration of traditional water treatment and granular activated carbon; ii) integration of traditional water treatment and membrane filtration; iii) integration of traditional water treatment and, ozonation and granular activated carbon; iv) integration of pre-ozonation and traditional water treatment, ozonation and granular activated carbon.<br>II. Wastewater treatment:	[69,73,77,78,85,87]

		i) traditional activated sludge with extended aeration.	
Disinfection byproducts (DBPs)	3-Hydroxybenzoic (99-06-9), Benzoic acid (65-85-0), Bromodichloromethane (75-27-4), Bromoform (75-25-2), Dichloroacetamide (683-72-7), Fumaric (110-17-8), Glyoxal (107-22-2), Methyl-Glyoxal (78-98-8), N-nitrosodimethylamine (62-75-9), Phthalic (88-99-3), Propionaldehyde (123-38-6), Protocatechuic (99-50-3)	I. Water treatment: i) traditional water treatment (including coagulation, flocculation-sedimentation, filtration and chlorination); ii) integration of pre-ozonation and traditional water treatment; iii) integration of pre-ozonation and traditional water treatment, ozonation and granular activated carbon.<br>II. Wastewater reuse treatment: i) integration of ozonation and membrane filtration and UV/H_2O_2 advanced oxidation process; ii) integration of ozonation and biological activated carbon, membrane filtration and UV/H_2O_2 advanced oxidation process.	88-90
Personal care products (PCPs)	(5-Chloro-2H-benzotriazol-2-yl)-4-methyl-6-(2-methyl-2-propanyl) phenol (3896-11-5), 2-(2H-Benzotriazol-2-yl)-4-(2-methyl-2-propanyl) phenol (3147-76-0), 2-(2H-Benzotriazol-2-yl)-4,6-bis(2-methyl-2-butanyl) phenol (25973-55-1), 2-(2H-Benzotriazol-2-yl)-4,6-bis(2-phenyl-2-propanyl) phenol (70321-86-7), 2-(2H-Benzotriazol-2-yl)-4-methylphenol (2240-22-4), 2-(2H-Benzotriazol-2-yl)-6-sec-butyl-4-(2-methyl-2-propanyl) phenol (36437-37-3), 2,4-Di-tert-butyl-6-(5-chlorobenzotriazol-2-yl) phenol (3864-99-1), 3-(4-methylbenzylidene) camphor (36861-47-9), Benzophenone-3 (131-57-7), N, N-Diethyl-M-Toluamide (134-62-3), Triclosan (3380-34-5)	I. Water treatment: i) integration of traditional water treatment and granular activated carbon; ii) integration of traditional water treatment and ozonation, and activated carbon; iii) integration of pre-ozonation and traditional water treatment, ozonation and granular activated carbon.<br>II. Wastewater treatment: i) traditional activated sludge; ii) biological aerated filter-trickling filter; iii) membrane bioreactor; iv) traditional water treatment waste stabilization, lagoons-	73,78,81,83,85,91

		dissolved air flotation/filtration.

Note: The number of CECs in each category does not correspond to the number of circles displayed in Supplementary Fig. 5, due to the potential for individual CECs to be associated with multiple process configurations.

Supplementary Table 18 | Detailed environmental impacts of "RO (others)" depicted in Fig. 4.						
Environmental impact categories	Antiscalant	Citric acid	NaOH	Sodium sulfite	Incineration of waste membrane	Wastewater treatment of RO brines
Terrestrial acidification ($\times10^{-3}$ kg SO_2 eq)	7.93×10^{-3}	1.46×10^{-2}	3.79×10^{-3}	1.71×10^{-1}	1.13×10^{-1}	3.82×10^{-2}
Mineral consumption ($\times10^{-3}$ kg Cu eq)	1.02×10^{-2}	7.48×10^{-3}	3.70×10^{-3}	3.40×10^{-2}	7.74×10^{-4}	1.67×10^{-2}
Fossil fuel depletion ($\times10^{-1}$ kg oil eq)	1.26×10^{-2}	6.79×10^{-3}	2.16×10^{-3}	3.04×10^{-2}	4.95×10^{-2}	9.80×10^{-2}
Marine ecotoxicity ($\times10^{-2}$ kg 1,4-DCB)	1.30×10^{-2}	9.60×10^{-3}	6.23×10^{-3}	5.00×10^{-2}	4.03×10^{-4}	9.10×10^{-2}
Freshwater ecotoxicity ($\times10^{-2}$ kg 1,4-DCB)	9.11×10^{-3}	7.06×10^{-3}	4.46×10^{-3}	3.54×10^{-2}	7.67×10^{-5}	7.36×10^{-2}
Ozone depletion ($\times10^{-7}$ kg CFC11 eq)	9.44×10^{-3}	8.32×10^{-2}	1.28×10^{-2}	6.44×10^{-2}	6.42×10^{-2}	1.74×10^{-1}
Global warming (kg CO_2 eq)	3.02×10^{-3}	2.62×10^{-3}	8.90×10^{-4}	8.39×10^{-3}	-8.80×10^{-3}	3.49×10^{-2}
Freshwater eutrophication ($\times10^{-4}$ kg P eq)	9.49×10^{-3}	1.13×10^{-2}	7.65×10^{-3}	5.04×10^{-2}	5.75×10^{-5}	1.37×10^{-1}
Marine eutrophication ($\times10^{-5}$ kg N eq)	6.99×10^{-3}	1.59×10^{-1}	7.45×10^{-3}	4.05×10^{-2}	2.90×10^{-3}	1.01×10^{-1}
Terrestrial ecotoxicity (kg 1,4-DCB)	8.82×10^{-3}	5.72×10^{-3}	3.23×10^{-3}	3.11×10^{-2}	4.50×10^{-3}	1.37×10^{-2}

Supplementary References

1. Chen, J. et al. A machine-learning approach clarifies interactions between contaminants of emerging concern. *One Earth* **5**, 1239-1249 (2022).
2. Zijp, M. C., Van, d. L. H. Life Cycle Assessment of two drinking water production schemes (National Institute for Public Health and the Environment, 2015).
3. Sousi, M. et al. Comparing the bacterial growth potential of ultra-low nutrient drinking water assessed by growth tests based on flow cytometric intact cell count versus adenosine triphosphate. *Water Res.* **203**, 117506 (2021).
4. Sousi, M. et al. Multi-parametric assessment of biological stability of drinking water produced from groundwater: Reverse osmosis vs. conventional treatment. *Water Res.* **186**, 116317 (2020).
5. Fantke, P. et al. USEtox® 2.0 Documentation (Version 1). USEtox International Center http://usetox.org»(2017).
6. World Population Prospects 2019 Online Edition Rev. 1 (United Nations, Department of Economic and Social Affairs, 2019).
7. Bai, X. et al. Occurrence, distribution, and seasonality of emerging contaminants in urban watersheds. *Chemosphere* **200**, 133-142 (2018).
8. Kong, L. et al. Screening of 1300 organic micro-pollutants in groundwater from Beijing and Tianjin, North China. *Chemosphere* **165**, 221-230 (2016).
9. Gonzalez-Rey, M. et al. Occurrence of pharmaceutical compounds and pesticides in aquatic systems. *Mar. Pollut. Bull.* **96**, 384-400 (2015).
10. Yoon, Y. et al. Occurrence of endocrine disrupting compounds, pharmaceuticals, and personal care products in the Han River (Seoul, South Korea). *Sci. Total Environ.* **408**, 636-643 (2010).
11. Benotti, M. J. et al. Pharmaceuticals and Endocrine Disrupting Compounds in US Drinking Water. *Environ. Sci. Technol.* **43**, 597-603 (2009).
12. Sun, S. et al. Occurrence, spatial distribution, and seasonal variation of emerging trace organic pollutants in source water for Shanghai, China. *Sci. Total Environ.* **639**, 1-7 (2018).
13. Ribeiro, C. et al. Priority Substances and Emerging Organic Pollutants in Portuguese Aquatic Environment: A Review. *Rev. Environ. Contam. Toxicol.* **238**, 1-44 (2015).
14. Fingler, S. et al. Herbicide micropollutants in surface, ground and drinking waters within and near the area of Zagreb, Croatia. *Environ. Sci. Pollut. Res.* **24**, 1-14 (2016).
15. Masiá, A. et al. Pesticide monitoring in the basin of Llobregat River (Catalonia, Spain) and comparison with historical data. *Sci. Total Environ.* **503-504**, 58-68 (2015).
16. Ccanccapa, A. et al. Pesticides in the Ebro River basin: Occurrence and risk assessment. *Environ. Pollut.* **211**, 414-424 (2016).
17. Scott, P. D. et al. A National Survey of Trace Organic Contaminants in Australian Rivers. *J. Environ. Qual* **43**, 1702-1712 (2014).
18. Calderon-Preciado, D. et al. Screening of 47 organic microcontaminants in agricultural irrigation waters and their soil loading. *Water Res* **45**, 221-231 (2011).
19. Troger, R. et al. Micropollutants in drinking water from source to tap - Method development and application of a multiresidue screening method. *Sci. Total Environ.* **627**, 1404-1432 (2018).
20. You, L. et al. Investigation of pharmaceuticals, personal care products and endocrine disrupting chemicals in a tropical urban catchment and the influence of environmental factors. *Sci. Total Environ.* **536**, 955-963 (2015).
21. Esteban, S. et al. Analysis and occurrence of endocrine-disrupting compounds and estrogenic activity in

the surface waters of Central Spain. *Sci. Total Environ.* **466-467**, 939-951 (2014).
22. Li, B. et al. Occurrence and distribution of phthalic acid esters and phenols in Hun River Watersheds. *Environmental Earth Sciences* **73**, 5095-5106 (2015).
23. Wolschke, H. et al. Organophosphorus flame retardants and plasticizers in the aquatic environment: A case study of the Elbe River, Germany. *Environ Pollut* **206**, 488-493 (2015).
24. Sang, D., Kim et al. Occurrence and removal of pharmaceuticals and endocrine disruptors in South Korean surface, drinking, and waste waters. *Water Res.* **41**, 1013-1021 (2007).
25. Wu, C. et al. Occurrence of pharmaceuticals and personal care products and associated environmental risks in the central and lower Yangtze river, China. *Ecotoxicol. Environ. Saf.* **106**, 19-26 (2014).
26. Vulliet, E., Cren-Olive, C. Screening of pharmaceuticals and hormones at the regional scale, in surface and groundwaters intended to human consumption. *Environ. Pollut.* **159**, 2929-2934 (2011).
27. Gavrilescu, M. et al. Emerging pollutants in the environment: present and future challenges in biomonitoring, ecological risks and bioremediation. *New Biotechnol.* **32**, 147-156 (2015).
28. Paíga, P. et al. Presence of pharmaceuticals in the Lis river (Portugal): Sources, fate and seasonal variation. *Sci. Total Environ.* **573**, 164-177 (2016).
29. Lin, Y. C. et al. Occurrence of pharmaceuticals, hormones, and perfluorinated compounds in groundwater in Taiwan. *Environ. Monit. Assess.* **187**, 256 (2015).
30. Montes-Grajales, D. et al. Occurrence of personal care products as emerging chemicals of concern in water resources: A review. *Sci. Total Environ.* **595**, 601-614 (2017).
31. Yao, B. et al. Occurrence and indicators of pharmaceuticals in Chinese streams: A nationwide study. *Environ. Pollut.* **236**, 889-898 (2018).
32. Nödler, K. et al. Occurrence and fate of the angiotensin II receptor antagonist transformation product valsartan acid in the water cycle – A comparative study with selected β-blockers and the persistent anthropogenic wastewater indicators carbamazepine and acesulfame. *Water Res.* **47**, 6650-6659 (2013).
33. Kasprzyk-Hordern, B. et al. The occurrence of pharmaceuticals, personal care products, endocrine disruptors and illicit drugs in surface water in South Wales, UK. *Water Res.* **42**, 3498-3518 (2008).
34. Gracia-Lor, E. et al. Investigation of pharmaceutical metabolites in environmental waters by LC-MS/MS. *Environ. Sci. Pollut. Res.* **21**, 5496-5510 (2014).
35. Bu, Q. et al. Pharmaceuticals and personal care products in the aquatic environment in China: A review. *J. Hazard. Mater.* **262**, 189-211 (2013).
36. Birch, G. F. et al. Emerging contaminants (pharmaceuticals, personal care products, a food additive and pesticides) in waters of Sydney estuary, Australia. *Mar. Pollut. Bull.* **97**, 56-66 (2015).
37. Wu, M. et al. Occurrence and fate of psychiatric pharmaceuticals in the urban water system of Shanghai, China. *Chemosphere* **138**, 486-493 (2015).
38. Archer, E. et al. Pharmaceutical and personal care products (PPCPs) as endocrine disrupting contaminants (EDCs) in South African surface waters. *Water SA* **43**, 684-706 (2017).
39. Hu, X. L. et al. Occurrence of 25 pharmaceuticals in Taihu Lake and their removal from two urban drinking water treatment plants and a constructed wetland. *Environ. Sci. Pollut. Res.* **24**, 14889-14902 (2017).
40. Vieno, N. M. et al. Occurrence of pharmaceuticals in river water and their elimination a pilot-scale drinking water treatment plant. *Environ. Sci. Technol.* **41**, 5077-5084 (2007).
41. Yang, L. et al. Occurrence, distribution, and attenuation of pharmaceuticals and personal care products in the riverside groundwater of the Beiyun River of Beijing, China. *Environ. Sci. Pollut. Res.* **24**, 15838-15851 (2017).

42. Aydin, E., Talinli, I. Analysis, occurrence and fate of commonly used pharmaceuticals and hormones in the Buyukcekmece Watershed, Turkey. *Chemosphere* **90**, 2004-2012 (2013).
43. Togola, A., Budzinski, H. Multi-residue analysis of pharmaceutical compounds in aqueous samples. *J. Chromatogr. A* **1177**, 150-158 (2008).
44. Peng, F.-J. et al. Occurrence and ecological risk assessment of emerging organic chemicals in urban rivers: Guangzhou as a case study in China. *Sci. Total Environ.* **589**, 46-55 (2017).
45. Campanha, M. B. et al. A 3-year study on occurrence of emerging contaminants in an urban stream of São Paulo State of Southeast Brazil. *Environ. Sci. Pollut. Res.* **22**, 7936 (2015).
46. Ma, R. et al. Characterization of pharmaceutically active compounds in Dongting Lake, China: Occurrence, chiral profiling and environmental risk. *Sci. Total Environ.* **557-558**, 268-275 (2016).
47. Nkoom, M. et al. Occurrence and ecological risk assessment of pharmaceuticals and personal care products in Taihu Lake, China: a review. *Environ. Sci.: Processes Impacts* **20**, 1640-1648 (2018).
48. Tamtam, F. et al. Occurrence and fate of antibiotics in the Seine River in various hydrological conditions. *Sci. Total Environ.* **393**, 84-95 (2008).
49. Xu, W. et al. Antibiotics in riverine runoff of the Pearl River Delta and Pearl River Estuary, China: Concentrations, mass loading and ecological risks. *Environ. Pollut.* **182**, 402-407 (2013).
50. Regnery, J. et al. Start-up performance of a full-scale riverbank filtration site regarding removal of DOC, nutrients, and trace organic chemicals. *Chemosphere* **127**, 136-142 (2015).
51. Heberer, T. et al. Field studies on the fate and transport of pharmaceutical residues in bank filtration. *Ground Water Monit. Rem.* **24**, 70-77 (2004).
52. Glorian, H. et al. Water Quality Monitoring in Northern India for an Evaluation of the Efficiency of Bank Filtration Sites. *Water* **10** (2018).
53. Hoppe-Jones, C. et al. Attenuation of total organic carbon and unregulated trace organic chemicals in US riverbank filtration systems. *Water Res.* **44**, 4643-4659 (2010).
54. Sudhakaran, S. et al. Appropriate drinking water treatment processes for organic micropollutants removal based on experimental and model studies — A multi-criteria analysis study. *Sci. Total Environ.* **442**, 478-488 (2013).
55. Heberer, T. et al. From municipal sewage to drinking water: fate and removal of pharmaceutical residues in the aquatic environment in urban areas. *Water Sci. Technol.* **46**, 81-88 (2002).
56. Schmidt, C. K. et al. Characteristics and evaluation of natural attenuation processes for organic micropollutant removal during riverbank filtration. *Water Sci. Technol. Water Supply.* **7**, 1-7 (2007).
57. Nagy-Kovacs, Z. et al. Behavior of Organic Micropollutants During River Bank Filtration in Budapest, Hungary. *Water* **10**, 1861 (2018).
58. Van Driezum, I. H. et al. Spatiotemporal resolved sampling for the interpretation o micropollutant removal during riverbank filtration. *Sci. Total Environ.* **649**, 212-223 (2019).
59. Kovacevic, S. et al. Occurrence and behavior of selected pharmaceuticals during riverbank filtration in The Republic of Serbia. *Environ. Sci. Pollut. Res.* **24**, 2075-2088 (2017).
60. Heberer, T. et al. Behaviour and redox sensitivity of antimicrobial residues during bank filtration. *Chemosphere* **73**, 451-460 (2008).
61. Rodriguez-Mozaz, S. et al. Pharmaceuticals and pesticides in reclaimed water: Efficiency assessment of a microfiltration–reverse osmosis (MF–RO) pilot plant. *J. Hazard. Mater.* **282**, 165-173 (2015).
62. Taheran, M. et al. Membrane processes for removal of pharmaceutically active compounds (PhACs) from water and wastewaters. *Sci. Total Environ.* **547**, 60-77 (2016).
63. Comerton, A. M. et al. The rejection of endocrine disrupting and pharmaceutically active compounds by

NF and RO membranes as a function of compound and water matrix properties. *J. Membr. Sci.* **313**, 323-335 (2008).
64. Kim, S. D. et al. Occurrence and removal of pharmaceuticals and endocrine disruptors in South Korean surface, drinking, and waste waters. *Water Res.* **41**, 1013-1021 (2007).
65. Wintgens, T. et al. Emerging contaminants and treatment options in water recycling for indirect potable use. *Water Sci. Technol.* **57**, 99-107 (2008).
66. Gomez, V. et al. Determination and occurrence of organic micropollutants in reverse osmosis treatment for advanced water reuse. *Water Sci. Technol.* **66**, 61-71 (2012).
67. Lin, T. et al. Occurrence, removal and risk assessment of pharmaceutical and personal care products (PPCPs) in an advanced drinking water treatment plant (ADWTP) around Taihu Lake in China. *Chemosphere* **152**, 1-9 (2016).
68. Pojana, G. et al. Occurrence of environmentally relevant pharmaceuticals in Italian drinking water treatment plants. *Intern. J. Environ. Anal. Chem.* **91**, 537-552 (2011).
69. Bourgin, M. et al. Evaluation of a full-scale wastewater treatment plant upgraded with ozonation and biological post-treatments: Abatement of micropollutants, formation of transformation products and oxidation by-products. *Water Res*. **129**, 486-498 (2018).
70. Boleda, M. R. et al. Behavior of pharmaceuticals and drugs of abuse in a drinking water treatment plant (DWTP) using combined conventional and ultrafiltration and reverse osmosis (UF/RO) treatments. *Environ. Pollut.* **159**, 1584-1591 (2011).
71. Huerta-Fontela, M. et al. Occurrence and removal of pharmaceuticals and hormones through drinking water treatment. *Water Res.* **45**, 1432-1442 (2011).
72. Kyu, H. H. et al. Global, regional, and national disability-adjusted life-years (DALYs) for 359 diseases and injuries and healthy life expectancy (HALE) for 195 countries and territories, 1990-2017: a systematic analysis for the Global Burden of Disease Study 2017. *Lancet* **392**, 1859-1922 (2018).
73. Fajnorová, S. et al. Assessment of Full-Scale Indirect Potable Water Reuse in El Port de la Selva, Spain. *Water* **13**, 325 (2021).
74. Padhye, L. P. et al. Year-long evaluation on the occurrence and fate of pharmaceuticals, personal care products, and endocrine disrupting chemicals in an urban drinking water treatment plant. *Water Res.* **51**, 266-276 (2014).
75. De Caroli Vizioli, B. et al. Atrazine and its degradation products in drinking water source and supply: Risk assessment for environmental and human health in Campinas, Brazil. *Chemosphere* **336**, 139289 (2023).
76. Jemal, T. et al. Effect of conventional and household water treatment technologies on the removal of pesticide residues in drinking water, Jimma town, Southwestern, Ethiopia. *PLoS One* **18**, e0288086 (2023).
77. Xu, C. et al. Occurrence, impact variables and potential risk of PPCPs and pesticides in a drinking water reservoir and related drinking water treatment plants in the Yangtze Estuary. *Environ. Sci.: Processes Impacts* **20**, 1030-1045 (2018).
78. Borrull, J. et al. Presence, behaviour and removal of selected organic micropollutants through drinking water treatment. *Chemosphere* **276**, 130023 (2021).
79. Tröger, R. et al. What's in the water? – Target and suspect screening of contaminants of emerging concern in raw water and drinking water from Europe and Asia. *Water Res.* **198**, 117099 (2021).
80. Elfikrie, N. et al. Occurrence of pesticides in surface water, pesticides removal efficiency in drinking water treatment plant and potential health risk to consumers in Tengi River Basin, Malaysia. *Sci. Total Environ.* **712**, 136540 (2020).
81. Pan, C.-G. et al. Perfluoroalkyl substances (PFASs) in wastewater treatment plants and drinking water

treatment plants: Removal efficiency and exposure risk. *Water Res.* **106**, 562-570 (2016).
82. Tran, N. H., Gin, K. Y.-H. Occurrence and removal of pharmaceuticals, hormones, personal care products, and endocrine disrupters in a full-scale water reclamation plant. *Sci. Total Environ.* **599-600**, 1503-1516 (2017).
83. Cao, J. et al. Fate of typical endocrine active compounds in full-scale wastewater treatment plants: Distribution, removal efficiency and potential risks. *Bioresour. Technol.* **310**, 123436 (2020).
84. Liu, Y.-S. et al. Occurrence and removal of benzotriazoles and ultraviolet filters in a municipal wastewater treatment plant. *Environ. Pollut.* **165**, 225-232 (2012).
85. Andresen, J., Bester, K. Elimination of organophosphate ester flame retardants and plasticizers in drinking water purification. *Water Res.* **40**, 621-629 (2006).
86. Jiang, X. et al. Seasonal and spatial variations of pharmaceuticals and personal care products occurrence and human health risk in drinking water - A case study of China. *Sci. Total Environ.* **694**, 133711 (2019).
87. Simazaki, D. et al. Occurrence of selected pharmaceuticals at drinking water purification plants in Japan and implications for human health. *Water Res.* **76**, 187-200 (2015).
88. Gao, P. et al. Occurrence of pharmaceuticals in a municipal wastewater treatment plant: Mass balance and removal processes. *Chemosphere* **88**, 17-24 (2012).
89. Zhong, X. et al. Seasonal evaluation of disinfection by-products throughout two full-scale drinking water treatment plants. *Chemosphere* **179**, 290-297 (2017).
90. Zeng, T. et al. N-Nitrosamines and halogenated disinfection byproducts in U.S. Full Advanced Treatment trains for potable reuse. *Water Res.* **101**, 176-186 (2016).
91. Serrano, M. et al. Seasonal evaluation of the presence of 46 disinfection by-products throughout a drinking water treatment plant. *Sci. Total Environ.* **517**, 246-258 (2015).
92. Zhao, X. et al. Occurrence and fate of benzotriazoles UV filters in a typical residential wastewater treatment plant in Harbin, China. *Environ. Pollut.* **227**, 215-222 (2017).
