# Supplementary material for: Leveraging the water-environment-health nexus to characterize sustainable water purification solutions
Source: Nat Commun. 2025 Feb 2;16:1269. doi: 10.1038/s41467-025-56656-6 (PMC11788440; doi:10.1038/s41467-025-56656-6)
Supplement: Supplementary file 2 — Description of Additional Supplementary Files [file 41467_2025_56656_MOESM2_ESM.pdf]

Description of Additional Supplementary Files

File Name: Supplementary Data 1
Description: Electricity generation mix in different world contexts with resultant effects on environmental performance of the RBF-RO system.
